# ﻿Integrative taxonomy delimits and diagnoses cryptic arboreal species of the *Cyrtodactylusbrevipalmatus* group (Squamata, Gekkonidae) with descriptions of four new species from Thailand

**DOI:** 10.3897/zookeys.1129.90535

**Published:** 2022-11-15

**Authors:** L. Lee Grismer, Anchalee Aowphol, Siriporn Yodthong, Natee Ampai, Korkhwan Termprayoon, Akrachai Aksornneam, Attapol Rujirawan

**Affiliations:** 1 Herpetology Laboratory, Department of Biology, La Sierra University, 4500 Riverwalk Parkway, Riverside, California 92505, USA La Sierra University Riverside United States of America; 2 Department of Herpetology, San Diego Natural History Museum, PO Box 121390, San Diego, California, 92112, USA Department of Herpetology, San Diego Natural History Museum San Diego United States of America; 3 Animal Systematics and Ecology Speciality Research Unit, Department of Zoology, Faculty of Science, Kasetsart University, Bangkok 10900, Thailand Kasetsart University Bangkok Thailand; 4 Department of Biology, Faculty of Science, Thaksin University, Pa Phayom, Phattalung 93210, Thailand Thaksin University Phattalung Thailand; 5 Department of Biology, Faculty of Science, Srinakharinwirot University, Bangkok 10110, Thailand Srinakharinwirot University Bangkok Thailand

**Keywords:** geckos, genetics, morphology, Southeast Asia, taxonomy

## Abstract

Species delimitation and species diagnosis must remain separate operations to avoid constructing taxonomies comprised of non-monophyletic species based on morphological similarity as opposed to phylogenetic propinquity. This is particularly true for highly specialized species such as the range-restricted upland taxa in the *Cyrtodactylusbrevipalmatus* group of Indochina where strong selection pressure for an arboreal lifestyle has contributed to morphologically similar but distantly related species. This in turn, has resulted in a history of erroneous taxonomies that have actually obscured rather than revealed the diversity within this group. A Bayesian phylogeny of the *C.brevipalmatus* group recovered at least 15 putative species-level lineages, at least seven of which are undescribed, and of which four are described herein. A total evidence morphological data set comprised of 16 normalized morphometric, 15 meristic, and seven categorical characters scored across 51 individuals was subjected to a multiple factor analysis (MFA) and an analysis of variance (ANOVA) to diagnose the putative species. These new species descriptions contribute to focusing attention to the unrealized diversity in upland tropical ecosystems, which due to climate change, are some of the most impearled ecosystems on the planet. Thus, it is paramount that taxonomies do not conflate species identities and underrepresent true diversity.

## ﻿Introduction

A cornerstone of biodiversity conservation is a phylogenetic-based taxonomy where the names of the component species are consistent with the patterns and processes by which they evolved. Taxonomies constructed from paraphyletic or polyphyletic species misrepresent history, thus obscuring true diversity and potentially countermanding the effectiveness of conservation efforts. Before a newly discovered population can be given a new name, or the name of an existing species be successfully challenged, it should be properly delimited and diagnosed. Delimiting and diagnosing species are independent operations used together to construct taxonomies that reflect, and are consistent with evolutionary history. Unfortunately, these two operations are often conflated when analyses to diagnose species, which are most often rooted in morphological similarity, are equated with analyses to delimit species, which are rooted in phylogenetic propinquity. The unfortunate consequence of this is that taxonomies may be constructed using non-monophyletic species, thus obscuring rather than revealing the group’s actual diversity. This is especially true for taxonomies comprised of highly specialized cryptic species where, in the absence of a phylogeny, morphological convergence can be mistaken for common ancestry (see Grismer 2008; Grismer et al. 2020, 2022). Despite methodological improvements over the years that have increased the efficacy of both delimitation and diagnostic procedures, they have had little effect on operational decisions to not conflate them.

**Figure 1.**
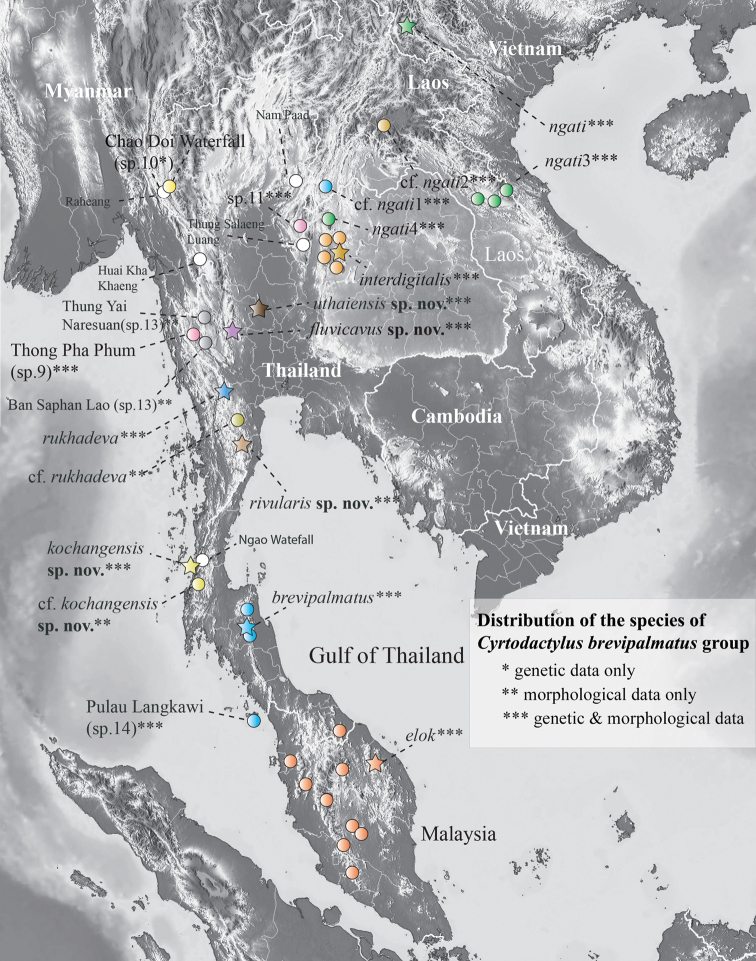
Distribution of nominal species and unnamed populations and specimens of the *Cyrtodactylusbrevipalmatus* group. Stars denote type localities. White circles are literature localities from which specimens were not examined and remain unidentified. Locality data for all material examined is in Table 1.

Convergent morphology results from the specific resource requirements that necessitate a particular functional morphology (Ricklefs 2012; Dehling et al. 2016; Baulechner et al. 2020). In such cases, phylogenetic analysis becomes paramount to disentangle convergence from common ancestry in order to construct proper phylogenetic taxonomies. This is especially true of clades containing highly specialized species such as the *Cyrtodactylusbrevipalmatus* group (sec. Grismer et al. 2021a, b, c). This group currently contains four nominal species that range from northern Vietnam and Laos, southward through Thailand to southern Peninsular Malaysia (Fig. 1). Nearly all members of this adaptive radiation are highly specialized for an arboreal lifestyle and all have a prehensile tail carried in a tightly coiled position, a cryptic color pattern of different shades of brown that closely match the substrate of their microhabitat, and generally slow, deliberate “chameleon-like” movements. Presumably, the selection pressures for such a specialized lifestyle have contributed to morphological convergence within this relatively small group (Grismer et al. 2022). Thus, as demonstrated by Grismer (2008) and Grismer et al. (2021c, 2022), previous morphology-based operations intending to delimit species resulted in reoccurring taxonomies composed of non-monophyletic species (Smith 1935; Welch et al. 1990; Ulber 1993; Manthey and Grossmann 1997; Stuart 1999; Nabhitabhata et al. 2004; Nabhitabhata and Chan-ard 2005; Pauwels and Chan-ard 2006; Ellis and Pauwels 2012) that underrepresented the group’s diversity. Grismer et al. (2021c, 2022) were the first to employ phylogenetic analyses to the *brevipalmatus* group to delimit species and statistically based morphological analyses to diagnose those species. As such, by treating these analyses as independent operations and their results as corroborating evidence, a revised taxonomy consistent with the group’s evolutionary history was disentangled from a convergence-based taxonomy.

The phylogenetic-based taxonomy of Grismer et al. (2022) is expanded here with significantly increased morphological and genetic sampling from new localities in Thailand at Ko (= island) Chang (ZMKU R 00945) and Khlong Nakha Wildlife Sanctuary (THNHM 01667), Ranong Province; Hua Hin, Prachuap Khiri Khan Province (ZMKU R 00946–47); Si Sawat (ZMKU R 00958–64) and Thong Pha Phum (ZMKU R 00950–57), Kanchanaburi Province; and an isolated population from a small hilly region at Lan Sak, Uthai Thani Province (ZMKU R 00949) in the Chao Phraya River Basin. In this analysis, we discuss the high degree of morphological similarity throughout the phylogeny despite the relatively high degrees of genetic divergence. The analyses also recovered the existence of 4–10 new populations we hypothesize to be distinct species, four of which are described herein.

## ﻿Materials and methods

### ﻿Species delimitation

The general lineage concept (GLC: de Queiroz 2007) adopted herein proposes that a species constitutes a population of organisms evolving independently from other such populations owing to a lack of, or limited gene flow. By “independently,” it is meant that new mutations arising in one species cannot spread readily into another species (Barraclough et al. 2003; de Queiroz 2007). Under the GLC implemented herein, molecular phylogenies recovered monophyletic mitochondrial lineages of individuals (populations) that were used to develop initial species-level hypotheses, the grouping stage of Hillis (2019). Discrete color pattern data and univariate and multivariate analyses of morphological data were then used to search for characters and morphospatial patterns consistent with the tree-designated species-level hypotheses, the construction of boundaries representing the hypothesis-testing step of Hillis (2019), –thus providing independent diagnoses to complement the molecular analyses. In this way, delimiting (phylogeny) and diagnosing (taxonomy) species are not conflated (Frost and Hillis 1990; Frost and Kluge 1994; Hillis 2019).

Species boundaries were cross-checked using a Bayesian Poisson Tree Process for species delimitation (bPTP; Zhang et al. 2013), thus providing an independent framework to complement the empirically based thresholds of the morphological and molecular analyses. This method delimits species from single-locus gene trees with low population samples (Lin et al. 2018) by detecting genetic clustering beyond the expected levels of a null hypothesis which infers that all individuals of a population form a genetically, interacting nexus. A Markov Chain Monte Carlo (MCMC) was run for 10,000 generations on the bPTP web server at https://species.h-its.org/ptp/ and checked for convergence. Delimitation accuracy was based on the normalized mutual information criterion (NMI) (Vinh et al. 2010). The model relies on the prediction that independent evolution leads to the appearance of distinct genetic clusters, separated by relatively longer internal branches (Barraclough et al. 2003; Acinas et al. 2004). Such groups therefore diverge into discrete clades of genetic variation. These clades are then diagnosed by statistically defensible morphological analyses.

### ﻿Genetic data

Methods for DNA extraction, sequencing, and editing followed Grismer et al. (2022) and resulted in a 1,386 base pair segment of the mitochondrial NADH dehydrogenase subunit 2 gene (ND2) and adjacent tRNAs. All material examined is listed in Table 1 along with GenBank accession numbers for the new and published genetic materials.

**Table 1.** *Cyrtodactylus* specimens examined in this study. Institutional abbreviations follow Sabaj (2020) except that YC = Yodchaiy Chuaynkern from Department of Biology, Faculty of Science, Khon Kaen University, Khon Kaen, Thailand.

Species	Location	Catalog no.	GenBank no.
*C.brevipalmatus*	Thailand, no data	LSUHC 1899	not in tree
*C.brevipalmatus*	Thailand, Nakhon Si Thammarat Province, Nopphitam District, Khao Nan National Park, Huay Lak Protected Unit	THNHM 10670	not in tree
*C.brevipalmatus*	Thailand, Nakhon Si Thammarat Province, Lan Saka District, Khao Luang National Park	THNHM 14112	not in tree
*C.brevipalmatus*	Thailand, Nakhon Si Thammarat Province, Khao Ram Mt.	AUP-00573	OK626313
C.cf.brevipalmatus	Peninsular Malaysia, Kedah State, Pulau Langkawi, Gunung Raya	LSUHC 11788	not in tree
C.cf.brevipalmatus	Peninsular Malaysia, Kedah State, Pulau Langkawi, Gunung Raya	LSUHC 15076	not in tree
*C.elok*	Peninsular Malaysia, Pahang State, Fraser’s Hill, the Gap	ZRC 2.6091/LSUHC 6471	JQ889180
*C.elok*	Peninsular Malaysia, Negeri Sembilan State	LSUHC 8238	not in tree
*C.elok*	Peninsular Malaysia, Pahang State, near Cameron Highlands	LSUHC 12180	not in tree
*C.elok*	Peninsular Malaysia, Pahang State, near Cameron Highlands	LSUHC 12181	not in tree
*C.elok*	Malaysian pet trade, no data	ZMMU R-16144	not in tree
*Cyrtodactylusfluvicavus* sp. nov.	Thailand, Kanchanaburi Province, Si Sawat District, Khao Chot Subdistrict, Chaloem Rattanakosin National Park	ZMKU R 00958 paratype	OP620036
*Cyrtodactylusfluvicavus* sp. nov.	Thailand, Kanchanaburi Province, Si Sawat District, Khao Chot Subdistrict, Chaloem Rattanakosin National Park	ZMKU R 00959 holotype	OP620037
*Cyrtodactylusfluvicavus* sp. nov.	Thailand, Kanchanaburi Province, Si Sawat District, Khao Chot Subdistrict, Chaloem Rattanakosin National Park	ZMKU R 00960 paratype	OP620038
*Cyrtodactylusfluvicavus* sp. nov.	Thailand, Kanchanaburi Province, Si Sawat District, Khao Chot Subdistrict, Chaloem Rattanakosin National Park	ZMKU R 00961 paratype	OP620039
*Cyrtodactylusfluvicavus* sp. nov.	Thailand, Kanchanaburi Province, Si Sawat District, Khao Chot Subdistrict, Chaloem Rattanakosin National Park	ZMKU R 00962 paratype	OP620040
*Cyrtodactylusfluvicavus* sp. nov.	Thailand, Kanchanaburi Province, Si Sawat District, Khao Chot Subdistrict, Chaloem Rattanakosin National Park	ZMKU R 00963 paratype	OP620041
*Cyrtodactylusfluvicavus* sp. nov.	Thailand, Kanchanaburi Province, Si Sawat District, Khao Chot Subdistrict, Chaloem Rattanakosin National Park	ZMKU R 00964 paratype	OP620042
*C.interdigitalis*	Thailand, Phetchabun Province, Nam Nao National Park, Tham Yai Nam Nao	THNHM 20226 paratype	not in tree
*C.interdigitalis*	Thailand, Phetchabun Province, Nam Nao National Park, Tham Yai Nam Nao	THNHM 20227 paratype	not in tree
*C.interdigitalis*	Thailand, Phetchabun Province, Nam Nao National Park, Tham Yai Nam Nao	THNHM 20228 paratype	not in tree
*C.interdigitalis*	Thailand, Phetchabun Province, Nam Nao National Park, Tham Yai Nam Nao	THNHM 20229 paratype	not in tree
*C.interdigitalis*	Thailand, Phetchabun Province, Nam Nao National Park, Tham Yai Nam Nao	YC000952	ON055281
*Cyrtodactyluskochangensis* sp. nov.	Thailand, Ranong Province, Mueng Ranong District, Ko Phayam Subdistrict, Ko Chang	ZMKU R 00945 holotype	OP620023
C.cf.kochangensis sp. nov.	Thailand, Ranong Province, Khlong Nakha Wildlife Sanctuary	THNHM 01667	not in tree
*C.ngati*	Vietnam, Dien Bien Province, Dien Bien District, Pa Thom Commune, Pa Xa Lao Village, karst forest near Pa Thom Cave	HNUE-R00111 holotype	ON411220
*C.ngati*	Vietnam, Dien Bien Province, Dien Bien District, Pa Thom Commune, Pa Xa Lao Village, karst forest near Pa Thom Cave	HNUE-R00112 paratype	not in tree
*C.ngati*	Vietnam, Dien Bien Province, Dien Bien District, Pa Thom Commune, Pa Xa Lao Village, karst forest near Pa Thom Cave	IEBR 4829 paratype	OK626318
*C.ngati*	Vietnam, Dien Bien Province, Dien Bien District, Pa Thom Commune, Pa Xa Lao Village, karst forest near Pa Thom Cave	VNUF R.2020.12 paratype	OK626319
*C.ngati*3	Laos, Khammouane Province	VNUF R.2014.50	ON411221
*C.ngati*3	Laos, Khammouane Province, Phou Hin Poun National Biodiversity Conservation Area	FMNH 255454	JQ889181
*C.ngati*3	Laos, Khammouane Province, Phou Hin Poun National Biodiversity Conservation Area	FMNH 270492	OK626315
*C.ngati*3	Laos, Khammouane Province, Phou Hin Poun National Biodiversity Conservation Area	FMNH 270493	not in tree
*C.ngati*4	Thailand, Loei Province, Nam San Noi River, Phu Luang Wildlife Sanctuary	FMNH 265806	JX519471
C.cf.ngati1	Laos, Xaignabouli Province, Ban Pha Liep, Houay Liep Stream	NCSM 79472	OK626316
C.cf.ngati2	Laos, Vientiane Province	ZMMU R-14917	not in tree
C.cf.ngati2	Laos, Vientiane Province, tributary of Nam Pha River, Houay Wan Stream	NCSM 80100	OK626317
*Cyrtodactylusrivularis* sp. nov.	Thailand, Prachuap Khiri Khan Province, Hua Hin District, Huai Sat Yai Subdistrict, Kaeng Krachan National Park, Pa La-U Waterfall	ZMKU R 00946 paratype	OP620024
*Cyrtodactylusrivularis* sp. nov.	Thailand, Prachuap Khiri Khan Province, Hua Hin District, Huai Sat Yai Subdistrict, Kaeng Krachan National Park, Pa La-U Waterfall	ZMKU R 00947 holotype	OP620025
*C.rukhadeva*	Thailand, Ratchaburi Province, Suan Phueng District, Khao Laem Mountain	ZMMU R-16851 holotype	OK626320
*C.rukhadeva*	Thailand, Ratchaburi Province, Suan Phueng District, Hoop Phai Tong	ZMMU R-16852 paratype	not in tree
*C.rukhadeva*	Thailand, Ratchaburi Province, Suan Phueng District, Suan Phueng Subdistrict	ZMKU R 00948	OP620026
C.cf.rukhadeva	Thailand, Phetchaburi Province, Kaeng Krachan National Park	THNHM 01807	not in tree
C.cf.rukhadeva	Thailand, Phetchaburi Province, Kaeng Krachan National Park	THNHM 03251	not in tree
C.cf.rukhadeva	Thailand, Phetchaburi Province, Kaeng Krachan National Park	THNHM 03252	not in tree
C.cf.rukhadeva	Thailand, Phetchaburi Province, Kaeng Krachan National Park	THNHM 03253	not in tree
C.cf.rukhadeva	Thailand, Phetchaburi Province, Kaeng Krachan National Park	THNHM 03254	not in tree
C.cf.rukhadeva	Thailand, Phetchaburi Province, Kaeng Krachan National Park	THNHM 24622	not in tree
C.cf.rukhadeva	Thailand, Phetchaburi Province, Kaeng Krachan National Park	THNHM 24838	not in tree
*Cyrtodactylusuthaiensis* sp. nov.	Thailand, Uthai Thani Province, Lan Sak District, Thung Na Ngam Subdistrict	ZMKU R 00949 holotype	OP620027
*C.* sp.9	Thailand, Kanchanaburi Province, Thong Pha Phum District, Thong Pha Phum National Park	AUP-01715	MT468909
*C.* sp.9	Thailand, Kanchanaburi Province, Thong Pha Phum District, Pilok Subdistrict, Thong Pha Phum National Park	ZMKU R 00950	OP620028
*C.* sp.9	Thailand, Kanchanaburi Province, Thong Pha Phum District, Pilok Subdistrict, Thong Pha Phum National Park	ZMKU R 00951	OP620029
*C.* sp.9	Thailand, Kanchanaburi Province, Thong Pha Phum District, Pilok Subdistrict, Thong Pha Phum National Park	ZMKU R 00952	OP620030
*C.* sp.9	Thailand, Kanchanaburi Province, Thong Pha Phum District, Pilok Subdistrict, Thong Pha Phum National Park	ZMKU R 00953	OP620031
*C.* sp.9	Thailand, Kanchanaburi Province, Thong Pha Phum District, Pilok Subdistrict, Thong Pha Phum National Park	ZMKU R 00954	OP620032
*C.* sp.9	Thailand, Kanchanaburi Province, Thong Pha Phum District, Pilok Subdistrict, Thong Pha Phum National Park	ZMKU R 00955	OP620033
*C.* sp.9	Thailand, Kanchanaburi Province, Thong Pha Phum District, Pilok Subdistrict, Thong Pha Phum National Park	ZMKU R 00956	OP620034
*C.* sp.9	Thailand, Kanchanaburi Province, Thong Pha Phum District, Pilok Subdistrict, Thong Pha Phum National Park	ZMKU R 00957	OP620035
*C.* sp.10	Thailand, Tak Province, Tha Song Yang District, Mae Moei National Park, Chao Doi Waterfall	AUP-00680	MT468902
*C.* sp.11 (C.cf.interdigitalis)	Thailand, Phitsanulok Province, Phu Hin Rong Kla National Park	ZMMU R-16492	MW792061
*C.* sp.13	Thailand, Tak Province, Umphang District, Thung Yai Naresuan Wildlife Sanctuary	THNHM 00104	not in tree
*C.* sp.13	Thailand, Kanchanaburi Province, Thong Pha Phum District, Ban Saphan Lao	THNHM 27821	not in tree
*C.* sp.14 (C.cf.brevipalmatus)	Peninsular Malaysia, Kedah State, Pulau Langkawi, Gunung Raya	USMHC 2555	OK626314

### ﻿Morphological data

Morphological data included both meristic and morphometric characters. To reduce the degree of researcher bias, data were taken using the protocol of Le et al. (2021) and Grismer et al. (2022) and where possible, double checked by LLG using high resolution digital photographs and/or the actual specimens. All data were taken on the left side of the body (when possible) and measured to the nearest 0.1 mm using digital calipers under a Nikon SMZ745 stereomicroscope. Morphometric data taken were: snout-vent length **(SVL)**, taken from the tip of the snout to the vent; tail length **(TL)**, taken from the vent to the tip of the tail–original or partially regenerated; tail width **(TW)**, taken at the base of the tail immediately posterior to the postcloacal swelling; humeral length **(HumL)**, taken from the proximal end of the humerus at its insertion point in the glenoid fossa to the distal margin of the elbow while flexed 90°; forearm length **(ForL)**, taken on the ventral surface from the posterior margin of the elbow while flexed 90° to the inflection of the flexed wrist; femur length **(FemL)**, taken from the proximal end of the femur at its insertion point in the acetabulum to the distal margin of the knee while flexed 90°; tibia length **(TibL)**, taken on the ventral surface from the posterior margin of the knee while flexed 90° to the base of the heel; axilla to groin length **(AG)**, taken from the posterior margin of the forelimb at its insertion point on the body to the anterior margin of the hind limb at its insertion point on the body; head length **(HL)**, the distance from the posterior margin of the retroarticular process of the lower jaw to the tip of the snout; head width **(HW)**, measured at the angle of the jaws; head depth **(HD)**, the maximum height of head measured from the occiput to base of the lower jaw posterior to the eyes; eye diameter **(ED)**, the greatest horizontal diameter of the eye-ball; eye to ear distance **(EE)**, measured from the anterior edge of the ear opening to the posterior edge of the bony orbit; eye to snout distance or snout length **(ES)**, measured from anteriormost margin of the bony orbit to the tip of snout; eye to nostril distance **(EN)**, measured from the anterior margin of the bony orbit to the posterior margin of the external nares; interorbital distance **(IO)**, measured between the dorsomedial-most edges of the bony orbits; internarial distance **(IN)**, measured between the external nares across the rostrum; and ear length **(EL)**, greatest oblique length across the auditory meatus.

Meristic characters evaluated were the number of supralabial scales **(SL)**, counted from the largest scale at the corner of the mouth or posterior to the eye, to the rostral scale; infralabial scales **(IL)**, counted from termination of enlarged scales at the corner of the mouth to the mental scale; number of paravertebral tubercles **(PVT)** between the limb insertions counted in a straight line immediately left of the vertebral column; number of longitudinal rows of body tubercles **(LRT)** counted transversely across the body midway between the limb insertions from one ventrolateral body fold to the other; number of longitudinal rows of ventral scales **(VS)** counted transversely across the abdomen midway between limb insertions from one ventrolateral fold to the other; number of transverse rows of ventral scales **(VSM)** counted along the midline of the body from the postmentals to just anterior to the cloacal opening, stopping where the scales become granular; number of expanded subdigital lamellae on the fourth toe proximal to the digital inflection **(TL4E)** counted from the base of the first phalanx where it contacts the body of the foot to the largest scale on the digital inflection–the large contiguous scales on the palmar and plantar surfaces were not counted; number of small, generally unmodified subdigital lamellae distal to the digital inflection on the fourth toe **(TL4U)** counted from the digital inflection to the claw including the claw sheath; total number of subdigital lamellae **(TL4T)** beneath the fourth toe (i.e. TL4E + TL4U = TL4T); number of expanded subdigital lamellae on the fourth finger proximal to the digital inflection **(FL4E)** counted the same way as with TL4E; number of small generally unmodified subdigital lamellae distal to the digital inflection on the fourth finger **(FL4U)** counted the same way as with TL4U; total number of subdigital lamellae **(FL4T)** beneath the fourth toe (i.e. FL4E + FL4U = FL4T); total number of enlarged femoral scales **(FS)** from each thigh combined as a single metric; number of enlarged precloacal scales **(PCS)**;number of precloacal pores **(PP)** in males; the number of femoral pores **(FP)** in males from each thigh combined as a single metric; and the number of dark body bands **(BB)** between the dark band on the nape and the hind limb insertions on the body. A post-sacral or sacral band when present, was not counted. Categorical characters evaluated were the presence or absence of tubercles on the flanks **(FKT)**; single enlarged, unmodified, medial subcaudal scales **(SC2)**; enlarged medial subcaudals intermittent, medially furrowed, posteriorly emarginated **(SC3)**; slightly enlarged medial subcaudals **(SC1)**; large or small dorsolateral caudal tubercles **(DCT)** forming a narrow or wide ventrolateral caudal fringe **(VLF1)**; ventrolateral caudal fringe scales generally homogenous or not **(VLF2)**; and the cross-section of the tail round or square **(TLcross)**.

### ﻿Phylogenetic analyses

An input file implemented in BEAUti (Bayesian Evolutionary Analysis Utility) v. 2.4.6 was run in BEAST (Bayesian Evolutionary Analysis Sampling Trees) v. 2.4.6 (Drummond et al. 2012) on CIPRES (Cyberinfrastructure for Phylogenetic Research; Miller et al. 2010) in order to generate a BEAST phylogeny, employing a lognormal relaxed clock with unlinked site models and linked trees and clock models. bModelTest (Bouckaert and Drummond 2017), implemented in BEAST, was used to numerically integrate over the uncertainty of substitution models while simultaneously estimating the phylogeny using Markov chain Monte Carlo (MCMC). MCMC chains were run using a Yule prior for 40,000,000 generations and logged every 4,000 generations. The BEAST log file was visualized in Tracer v. 1.7.0 (Rambaut et al. 2018) to ensure effective sample sizes (ESS) were clearly above 200 for all parameters. A maximum clade credibility tree using mean heights at the nodes was generated using TreeAnnotator v. 1.8.0 (Rambaut and Drummond 2013) with a burn-in of 1,000 trees (10%). Nodes with Bayesian posterior probabilities (BPP) of 0.95 and above were considered strongly supported (Huelsenbeck et al. 2001; Wilcox et al. 2002). Uncorrected pairwise sequence divergences were calculated in MEGA 11 (Tamura et al. 2021) using the complete deletion option to remove gaps and missing data from the alignment prior to analysis.

### ﻿Statistical analyses

All statistical analyses were conducted using R Core Team (2018). Morphometric characters used in statistical analyses were SVL, AG, HumL, ForL, FemL, TibL, HL, HW, HD, ED, EE, ES, EN, IO, EL, and IN. Tail metrics were not used due to the high degree of incomplete sampling (i.e., regenerated, broken, or missing). In order to most successfully remove the effects of allometry (sec. Chan and Grismer 2022), size was normalized using the following equation: X_adj_ = log(X)-β[log(SVL)-log(SVL_mean_)], where X_adj_ = adjusted value; X = measured value; β = unstandardized regression coefficient for each population; and SVL_mean_ = overall average SVL of all populations (Thorpe 1975, 1983; Turan 1999; Lleonart et al. 2000), accessible in the R package *GroupStruct* (available at https://github.com/chankinonn/GroupStruct). The morphometrics of each species were normalized separately and then concatenated so as not to conflate potential intra- with interspecific variation (Reist 1986; McCoy et al. 2006). The juvenile *Cyrtodactylusngati* (HNUE-R00112) was removed from the data so as not to skew the normalization results. All data were scaled to their standard deviation to ensure they were analyzed on the basis of correlation and not covariance. Meristic characters analyzed were SL, IL, PVT, LRT, VS, VSM, TL4E, TL4U, TL4T, FL4E, FL4U, FL4T, FS, PCS, and BB. Precloacal and femoral pores were omitted from the multivariate analyses due to their absence in females. Categorical characters analyzed were DCT, VLF1, VLF2, TLcross, SC1, SC2, and SC3.

Small sample sizes (*N* = 1 or 2) for some of the species/populations precluded them from statistical analyses. A Levene’s test for the normalized morphometric and meristic characters was conducted to test for equal variances across all groups. Characters with equal variances were analyzed with an analysis of variance (ANOVA) and TukeyHSD *post hoc* test for mean comparisons involving more than three groups. Those with unequal variances were subjected to Welch’s *F*-test and Games-Howell *post hoc* test to test for mean comparisons involving more than three groups.

Morphospatial clustering and positioning among the species/populations was analyzed using multiple factor analysis (MFA) on a concatenated data set comprised of 15 meristic characters, 16 normalized morphometric characters, and seven categorical characters (Suppl. material 1). For this test, it was not necessary to remove populations represented by small sample sizes. The MFA was implemented using the *mfa* () command in the R package *FactorMineR* (Husson et al. 2017) and visualized using the *Factoextra* package (Kassambara and Mundt 2017). MFA is a global, unsupervised, multivariate analysis that incorporates qualitative and quantitative data (Pagès 2015) simultaneously, making it possible to analyze different data types in a nearly total evidence environment. In an MFA, each individual is described by a different set of variables (i.e., characters) which are structured into different data groups in a global data frame, in this case quantitative data (i.e., meristics and normalized morphometrics) and categorical data (i.e., scale, tubercle, and caudal morphology). In the first phase of the analysis, separate multivariate analyses are carried out for each set of variables: principal component analyses (PCA) for each quantitative data set and a multiple correspondence analysis (MCA) for the categorical data. The data sets are then normalized separately by dividing all their elements by the square root of their first eigenvalue. For the second phase of the analysis, these normalized data sets are concatenated into a single matrix for a final global PCA of the normalized data. Standardizing the data in this manner prevents one data type from overleveraging another. In other words, the normalization of the data in the first phase prevents data types with the greatest number of characters or the greatest amount of variation from outweighing other data types in the second phase. This way, the contributions of each data type to the overall variation in the data set are scaled to define the morphospatial distance between individuals as well as calculating each data type’s contribution to the overall variation in the analysis (Pagès 2015; Kassambara and Mundt 2017).

A non-parametric permutation multivariate analysis of variance (PERMANOVA) from the *vegan* package 2.5–3 in R (Oksanen et al. 2020) was used to determine if the centroid locations and group clustering of each species/population in the MFA were statistically different from one another (Skalski et al. 2018). The analysis was based on the calculation of a Gower (dis)similarity matrix using 50,000 permutations based on the loadings of the first four dimensions of the MFA. A pairwise *post hoc* test calculates the differences between all combinations of population pairs, generating a *p*-value, a Bonferroni-adjusted *p*-value, and a pseudo-*F* ratio (*F* statistic). A *p* < 0.05 is considered significant and larger *F* statistics indicate more pronounced group separation. A rejection of the null hypothesis (i.e., centroid positions and/or the spread of the data points [i.e. clusters] are no different from random) signifies a statistically significant difference between species/populations.

### ﻿Phylomorphospace

The BEAST phylogeny was projected onto the first two dimensions of the MFA plot using the *phylomorphospace* () command from the R package phytools (Revell 2012). This allows one to map the history of a group’s morphological diversification and infer the magnitude and direction of shape change along branches of the phylogeny (Sidlauskas 2008). Outgroups and species from the phylogenetic analyses not represented in the morphological dataset were excluded. To eliminate a potential bias caused by the highly derived *Cyrtodactyluselok* (see Grismer et al. 2022), it was not included.

Some of the populations examined had only genetic or morphological data. However, only phylogenetically delimited populations bearing morphological differences from other populations were described as new species. In some cases, populations represented by only morphological data but in close geographic proximity to named species from which they could not be separated morphologically, were considered *conferre* (cf.) to the named species pending further investigation.

## ﻿Results

### ﻿Phylogenetic analysis

**Figure 2.**
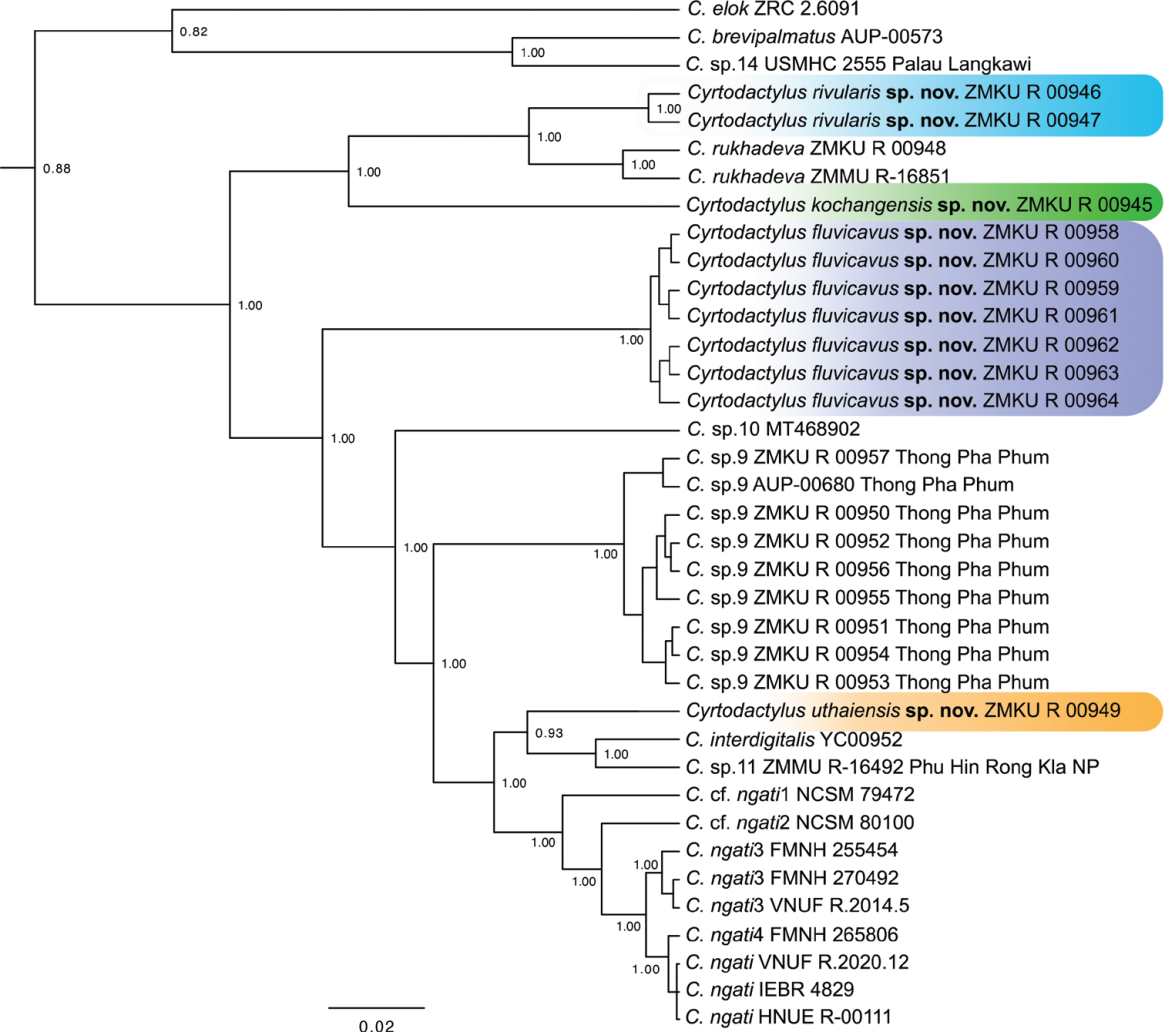
Maximum clade credibility BEAST phylogeny of the *Cyrtodactylusbrevipalmatus* group highlighting the new species described herein. Bayesian posterior probabilities (BPP) are listed at the nodes.

The BEAST analysis recovered 8–10 new species and two major clades within the *brevipalmatus* group: a weakly supported clade (BPP = 0.82) from the southernmost portion of the Thai-Malay Peninsula south of the Isthmus of Kra comprised of *Cyrtodactyluselok*, *C.brevipalmatus*, and *C.* sp.14 and a well-supported clade (1.00) comprised of all other species north of the Isthmus of Kra (Figs 1, 2). Within the latter, a well-supported (1.00) more exclusive clade containing the sister species from Hua Hin, Prachuap Khiri Khan (*Cyrtodactylusrivularis* sp. nov. [see below]) and *C.rukhadeva* with the Ko Chang population (*Cyrtodactyluskochangensis* sp. nov. [see below]) being sister to these. All relationships within this clade have strong nodal support (1.00) and its taxa occur only in the northernmost portion of the Thai-Malay Peninsula (Fig. 1). The population from Si Sawat (*Cyrtodactylusfluvicavus* sp. nov. [see below]), *C.* sp.10 from Chao Doi Waterfall, Mae Moei National Park, Tak Province, and *C.* sp.9 from Thong Pha Phum are sequentially related to a strongly supported (1.00) clade containing the remaining species. Within that latter clade, *C.interdigitalis* is the strongly supported (1.00) sister species to *C.* sp.11 from Phu Hin Rong Kla National Park and together they compose the well-supported (0.93) sister lineage to the Uthai Thani population (*Cyrtodactylusuthaiensis* sp. nov. [see below]). The remaining species all form a strongly supported (1.00) sequentially related clades with *C.ngati*.

Uncorrected pairwise sequence divergences within the *brevipalmatus* group range from 2.84–22.84% (Table 2). Ranges for those of the new species described below are as follows: *Cyrtodactylusrivularis* sp. nov. ranges from 3.61–21.81%, being most similar to *C.rukhadeva* and most distant to *C.elok*; *Cyrtodactylusfluvicavus* sp. nov. ranges from 9.55–20.26%, being most similar to the Thong Pha Phum population (*C.* sp.9) and most distant to *C.elok*; *Cyrtodactyluskochangensis* sp. nov. ranges from 12.00–20.90%, being most similar to *Cyrtodactylusrivularis* sp. nov. and most distant to *C.elok*; and *Cyrtodactylusuthaiensis* sp. nov. ranges from 5.81–21.16%, being most similar to C.cf.ngati1 and most distant to *C.elok*.

**Table 2.** Mean (minimum–maximum) percentages of uncorrected pairwise sequence divergence (*p*-distances) among the putative species of the *Cyrtodactylusbrevipalmatus* group based on 1,386 base pairs of mitochondrial NADH dehydrogenase subunit 2 gene (ND2) and adjacent tRNAs. Intraspecific *p*-distance are in bold font. n/a = data not applicable.

	*C.brevipalmatus*	C.cf.ngati1	C.cf.ngati2	*C.elok*	*Cyrtodactylusfluvicavus* sp. nov.	*C.interdigitalis*	*Cyrtodactyluskochangensis* sp. nov.	*C.ngati*, *C.ngati*3 and *C.ngati*4	*Cyrtodactylusrivularis* sp. nov.	*C.rukhadeva*	*C.* sp.9 (Thong Pha Phum)	*C.* sp.10	*C.* sp.11	*C.* sp.14	*Cyrtodactylusuthaiensis* sp. nov.
*C.brevipalmatus N* = 1	**n/a**														
C.cf.ngati1 *N* = 1	21.03	**n/a**													
C.cf.ngati2 *N* = 1	21.68	4.39	**n/a**												
*C.elok N* = 1	20.77	22.58	21.42	**n/a**											
*Cyrtodactylusfluvicavus* sp. nov. *N* = 7	18.86	10.64	11.02	20.15	**0.10**										
	(18.84–18.97)	(10.58–10.84)	(10.97–11.23)	(20.13–20.26)	**(0.00–0.26)**										
*C.interdigitalis N* = 1	20.77	6.97	9.16	22.84	12.02	**n/a**									
					(12.00–12.13)										
*Cyrtodactyluskochangensis* sp. nov. *N* = 1	19.35	14.58	14.71	20.90	12.31	15.23	**n/a**								
					(12.26–12.31)										
*C.ngati*, *C.ngati*3 and *C.ngati*4 *N* = 7	20.70	3.30	3.71	21.11	11.34	8.13	14.58	**0.84**							
	(20.65–20.90)	(2.84–4.00)	(3.35–4.26)	(20.90–21.42)	(11.10–11.87)	(7.74–8.65)	(14.45–14.84)	**(0.00–1.55)**							
*Cyrtodactylusrivularis* sp. nov. *N* = 2	20.00	15.87	15.03	21.61	12.57	15.48	12.26	15.03	**0.52**						
	(19.74–20.26)	(15.61–16.13)	(14.84–15.23)	(21.42–21.81)	(12.26–13.03)	(15.23–15.74)	(12.00–12.52)	(14.71–15.48)							
*C.rukhadeva N* = 2	20.65	15.42	15.48	21.61	12.25	16.00	13.10	15.23	4.65	**1.55**					
	(20.13–21.16)	(14.84–16.00)	(14.84–16.13)	(21.16–22.06)	(11.61–13.03)	(15.35–16.65)	(12.52–13.68)	(14.19–16.23)	(3.61–5.68)						
*C.* sp.9 (Thong Pha Phum) *N* = 9	20.34	7.93	9.51	22.02	9.75	8.96	13.22	8.81	13.12	13.25	**0.22**				
	(20.13–20.65)	(7.74–8.00)	(9.42–9.55)	(21.81–22.32)	(9.55–9.94)	(8.77–9.03)	(13.03–13.29)	(8.13–9.68)	(12.77–13.42)	(12.52–13.94)	**(0.00–0.52)**				
*C.* sp.10 *N* = 1	19.87	9.29	10.84	21.94	10.12	10.19	13.68	10.21	13.94	14.32	8.06	**n/a**			
					(10.06–10.32)			(10.06–10.45)	(13.68–14.19)	(13.68–14.97)	(7.87–8.13)				
*C.* sp.11 *N* = 1	20.39	7.23	8.90	22.19	11.12	3.87	14.58	8.28	15.35 1)	15.61	8.96	10.45	**n/a**		
					(11.10–11.23)			(8.00–8.65)	(15.10–15.6	(14.97–16.26)	(8.77–9.03)				
*C.* sp.14 *N* = 1	6.45	20.90	20.65	20.00	18.34	20.13	19.10	20.52	19.74	20.00	19.60	18.84	19.61	**n/a**	
					(18.32–18.45)			(20.26–20.65)	(19.48–20.00)	(19.48–20.52)	(19.48–19.87)				
*Cyrtodactylusuthaiensis* sp. nov. *N* = 1	19.74	5.81	8.13	21.16	10.12	7.1	13.94	6.97	13.94	13.94	7.80	8.39	6.58	19.48	**n/a**
					(10.06–10.32)			(6.58–7.61)	(13.68–14.19)	(13.29–14.58)	(7.61–7.87)				

### ﻿Bayesian Poisson Tree Process (bPTP)

The bPTP species delimitation analysis recovered 16 putative species within the *brevipalmatus* group with varying degrees of support (Table 3). The newly acquired material in this study recovered as distinct species are *Cyrtodactylusfluvicavus* sp. nov., *Cyrtodactyluskochangensis* sp. nov., *Cyrtodactylusrivularis* sp. nov., the holotype of *C.rukhadeva* (ZMMU R-16851), *C.rukhadeva* (ZMKU R 00948), *Cyrtodactylusuthaiensis* sp. nov., *C.* sp.9 from Thong Pha Phum, and *C.* sp.14 from Pulau Langkawi. However, *Cyrtodactylusrivularis* sp. nov. and *C.* sp.9 were not recovered with strong support (NMI 0.575 and 0.565, respectively) despite them being genetically distinct (Table 2) and morphologically diagnosable (Tables 4–6). The analysis also separated the two specimens of *C.rukhadeva* from the same locality which only differ by a genetic distance of 1.55% and are similar in morphology. Sukumaran and Knowles (2017) demonstrated that many species delimitation analyses recover clades not species and that a wider range of empirical data are necessary to interpret these boundaries (Coyne and Orr 1998; Feulner et al. 2007; Fontaneto et al. 2007; Knowles and Carstens 2007; Leach et al. 2009) as was done here (see below).

**Table 3.** Species delimited by the bPTP analysis.

Species	NMI
*C.brevipalmatus*	1
C.cf.ngati1	1
C.cf.ngati2	0.967
*C.elok*	1
*Cyrtodactylusfluvicavus* sp. nov.	0.957
*C.interdigitalis*	0.997
*Cyrtodactyluskochangensis* sp. nov.	1
*C.ngati*	0.841
*Cyrtodactylusrivularis* sp. nov.	0.575
*C.rukhadeva* ZMKU R 00948	0.947
*C.rukhadeva* ZMMU R-16851	0.947
*Cyrtodactylusuthaiensis* sp. nov	1
*C.* sp.9 (Thong Pha Phum)	0.565
*C.* sp.10	1
*C.* sp.11	0.997
*C.* sp.14	1

### ﻿Statistical analyses

**Figure 3.**
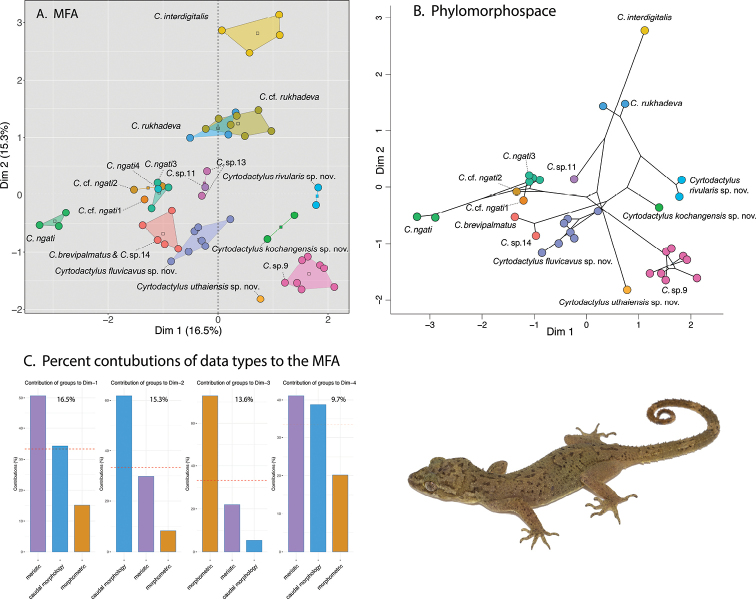
**A**MFA of the species-level lineages based on the BEAST phylogeny (Fig. 2) **B** phylomorphospace projection of the BEAST phylogeny **C** percent contribution of each data type to Dim 1–4 of the MFA. The percentage score at the top of each graph is the percent contribution of that dimension to the overall variation in the data set.

The ANOVA and TukeyHSD *post hoc* analyses of the adjusted morphometric and meristic characters were consistent with the phylogenetic and pairwise distance data in recovering a number of statistically significant differences between the *Cyrtodactylusfluvicavus* sp. nov. and *C.interdigitalis* as well as others (Table 6). *Cyrtodactylusfluvicavus* sp. nov. plotted separately in the MFA, only slightly overlapping with *C.brevipalmatus* (Fig. 3A). *Cyrtodactyluskochangensis* sp. nov., *Cyrtodactylusrivularis* sp. nov. and *Cyrtodactylusuthaiensis* sp. nov. plotted separately from each other and all other populations. A PERMANOVA analysis recovered several instances of various combinations of statistically significant differences among all the populations/species numbering more than two samples in regards to their clustering and centroid placement (Table 7). The contributions of each data type are shown in Fig. 3C.

### ﻿Morphological and phylogenetic discordance

The phylomorphospace analysis illustrates that the morphological similarities among the species/populations in the MFA are discordant with their phylogenetic placement in the tree (Fig. 3A, B). In other words, species/populations that are similar in morphology are not necessarily each other’s closest relatives. For example, *Cyrtodactylusinterdigitalis* is morphologically more similar to the distantly related *C.rukhadeva* than it is to *C.* sp.11 or *Cyrtodactylusuthaiensis* sp. nov. to which it is closely related. Similarly, *C.* sp.9 is morphologically similar to the distantly related *Cyrtodactyluskochangensis* sp. nov. than to the closely related clade containing *Cyrtodactylusuthaiensis* sp. nov., *C.interdigitalis*, *C.* sp.11, C.cf.ngati1, C.cf.ngati2, *C.ngati*3, *C.ngati*4, and *C.ngati*. Other examples of morphological and phylogenetic discordance can be seen by comparing Fig. 3A, B.

### ﻿Taxonomy

Given the phylogenetic delimitation of the Si Sawat, Prachuap Khiri Khan, Uthai Thani, and Ko Chang populations (Figs 2, 3), the statistically significant diagnostic placement of the Si Sawat population in morphospace (Fig. 3A; Table 7) and its statistically significant diagnostic morphological differences (Table 6), we describe them below as new species. The phylogenetic delimitation and statistically significant morphological differences among *C.* sp.9 from Thong Pha Phum and *C.* sp.11 from Phu Hin Rong Kla National Park will require descriptions at a future date.

#### ﻿ Cyrtodactylus fluvicavus sp. nov.

Taxon classificationAnimaliaSquamataGekkonidae

C909A4C7-64EF-5999-9A31-19E7EB8BF7B6

https://zoobank.org/B65469C6-55A1-401B-9EC3-FAFF151A2973

**Figure 4.**
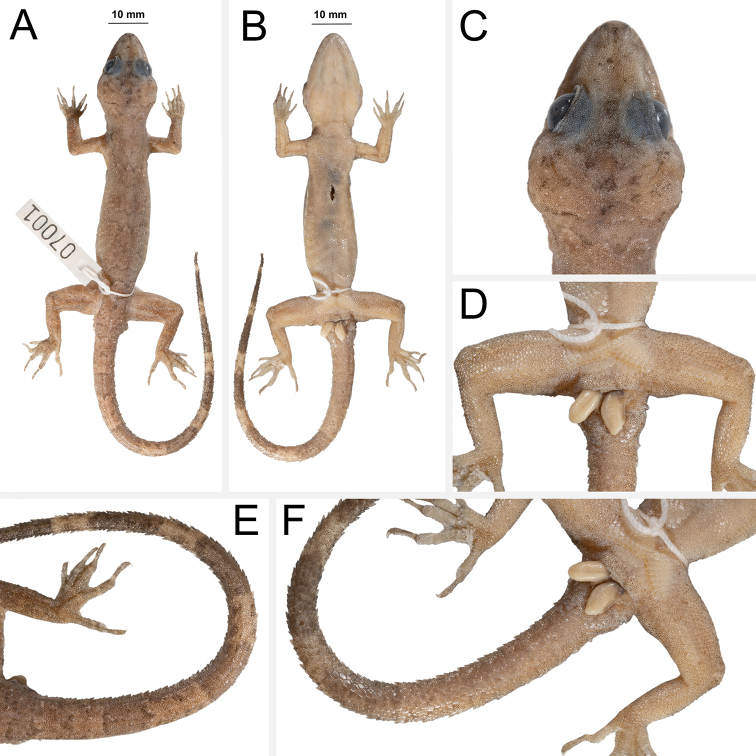
Holotype of *Cyrtodactylusfluvicavus* sp. nov. ZMKU R 00959 (field no. AA 07001) from Thailand, Kanchanaburi Province, Si Sawat District, Khao Chot Subdistrict, Chaloem Rattanakosin National Park **A** dorsal view **B** ventral view **C** dorsal view of head **D** ventral view of femoral and precloacal regions **E** dorsal view of tail **F** ventral view of tail.

**Figure 5.**
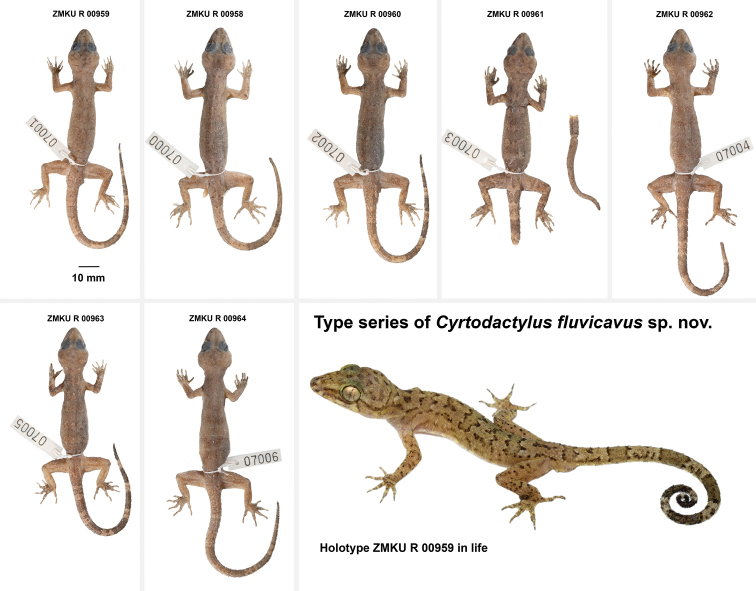
Type series of *Cyrtodactylusfluvicavus* sp. nov. from Thailand, Kanchanaburi Province, Si Sawat District, Khao Chot Subdistrict, Chaloem Rattanakosin National Park.

Figs 4 , 5 Suggested Common Name: Tham Than Lot Bent-toed Gecko

Cyrtodactylus sp. Yodthong, Rujirawan, Stuart, Grismer, Aksornneam, Termprayoon, Ampai & Aowphol, 2022: 161.

##### Holotype.

Adult male ZMKU R 00959 from Tham Than Lot Noi-Tham Than Lot Yai Nature Trail, Chaloem Rattanakosin National Park, Khao Chot Subdistrict, Si Sawat District, Kanchanaburi Province, Thailand (14.66930°N, 99.29060°E, 526 m a.s.l.), collected by Korkhwan Termprayoon, Akrachai Aksornneam, Natee Ampai, and Siriporn Yodthong on 20 April 2019.

##### Paratypes.

Adult males ZMKU R 00958 and ZMKU R 00960 and adult females ZMKU R 00961–64 bear the same collection site as the holotype.

##### Diagnosis.

*Cyrtodactylusfluvicavus* sp. nov. can be separated from all other species of the *brevipalmatus* group by the combination of having 11–13 supralabials, 9 or 10 infralabials, 26–30 paravertebral tubercles, 14–18 rows of longitudinally arranged tubercles, 30–39 transverse rows of ventrals, 154–175 longitudinal rows of ventrals, 9–11 expanded subdigital lamellae on the fourth toe, 10–13 unexpanded subdigital lamellae on the fourth toe, 19–22 total subdigital lamellae on the fourth toe; 7–9 expanded subdigital lamellae on the fourth finger, 9–11 unexpanded subdigital lamellae on the fourth finger, 17–19 total subdigital lamellae on the fourth finger; 9–12 total enlarged femoral scales, 8–11 total femoral pores in males; 14 or 15 precloacal pores in males; 14 or 15 enlarged precloacals; enlarged femorals and enlarged precloacals not continuous; proximal femorals less than one-half the size of the distal femorals; small tubercles on forelimbs and flanks; small dorsolateral caudal tubercles and narrow ventrolateral caudal fringe; ventrolateral caudal fringe composed scales of different size; tail circular in cross-section; slightly enlarged unpaired medial subcaudals not posteromedially furrowed; maximum SVL 78.2 mm; three dark transverse body bands (Tables 4–6).

##### Description of holotype

**(Fig. 4).** Adult male SVL 72.5 mm; head moderate in length (HL/SVL 0.28), width (HW/HL 0.70), depth (HD/HL 0.42), distinct from neck, triangular in dorsal profile; lores concave slightly anteriorly, weakly inflated posteriorly; prefrontal region concave; canthus rostralis rounded; snout elongate (ES/HL 0.42), rounded in dorsal profile; eye large (ED/HL 0.25); ear opening obliquely elongate, small; eye to ear distance greater than diameter of eye; rostral rectangular, divided by a deep furrow, bordered posteriorly by large left and right supranasals and one small azygous internasal, bordered laterally by first supralabials; external nares bordered anteriorly by rostral, dorsally by large supranasal, posteriorly by two smaller postnasals, bordered ventrally by first supralabial; 12R/12L rectangular supralabials, second through seventh supralabials nearly same size as first, then tapering abruptly below eye; 10R/10L infralabials tapering smoothly to just below and slightly past posterior margin of eye; scales of rostrum and lores flat to domed, larger than granular scales on top of head and occiput; scales of occiput intermixed with distinct, small tubercles; superciliaries subrectangular, largest dorsally; mental triangular, bordered laterally by first infralabials and posteriorly by large left and right trapezoidal postmentals contacting medially for 50% of their length posterior to mental; one row of slightly enlarged, elongate sublabials extending posteriorly to sixth(L) and fifth(R) infralabial; gular and throat scales small, granular, grading posteriorly into slightly larger, flatter, smooth, imbricate, pectoral and ventral scales.

Body relatively short (AG/SVL 0.46) with well-defined ventrolateral folds; dorsal scales small, granular interspersed with larger, conical, semi-regularly arranged, weakly keeled tubercles; tubercles extend from occipital region onto base of tail and slightly beyond as paravertebral rows; smaller tubercles extend anteriorly onto nape and occiput, diminishing in size anteriorly; approximately 17 longitudinal rows of tubercles at midbody; approximately 30 paravertebral tubercles; small tubercles on flanks; 34 longitudinal rows of flat, imbricate, ventral scales much larger than dorsal scales; 155 transverse rows of ventral scales; 15 large, pore-bearing, precloacal scales; no deep precloacal groove or depression; and two rows of post-precloacal scales on midline.

Forelimbs moderate in stature, relatively short (ForL/SVL 0.14); granular scales of forearm slightly larger than those on body, interspersed with large tubercles; palmar scales rounded, slightly raised; digits well-developed, relatively short, inflected at basal interphalangeal joints; digits narrower distal to inflections; subdigital lamellae wide, transversely expanded proximal to joint inflections, narrower transverse lamellae distal to joint inflections; claws well-developed, claw base sheathed by a dorsal and ventral scale; 8R/8L expanded and 10R/10L unexpanded lamellae beneath the fourth finger; hind limbs larger and thicker than forelimbs, moderate in length (TibL/SVL 0.16), covered dorsally by granular scales interspersed with moderately sized, conical tubercles dorsally and posteriorly and anteriorly by flat, slightly larger, subimbricate scales; ventral scales of thigh flat, subimbricate, larger than dorsals; subtibial scales flat, imbricate; one row of 5R/6L enlarged pore-bearing femoral scales not continuous with enlarged pore-bearing precloacal scales, terminating distally at knee; proximal femoral scales smaller than distal femorals, the former forming an abrupt union with much smaller, rounded, ventral scales of posteroventral margin of thigh; plantar scales flat; digits relatively long, well-developed, inflected at basal interphalangeal joints; 9R/9L wide, transversely expanded subdigital lamellae on fourth toe proximal to joint inflection extending onto sole, and 11R/11L unexpanded lamellae beneath the fourth toe; and claws well-developed, claw base sheathed by a dorsal and ventral scale.

Tail original, 97.6 mm long (TL/SVL 1.34), 5.2 mm in width at base, tapering to a point; sub-circular or nearly round in cross-section; dorsal scales flat, square bearing tubercles forming paravertebral rows and small tubercles forming a dorsolateral longitudinal row; slightly larger, posteriorly directed, semi-spinose tubercles forming narrow but distinct ventrolateral caudal fringe; larger scales of ventrolateral fringe occur at regular intervals; medial subcaudals slightly enlarged but not paired, distinctly enlarged single medial subcaudals absent; subcaudals, larger than dorsal caudals; base of tail bearing hemipenial swellings; 3R/2L conical postcloacal tubercles at base of hemipenial swellings; and postcloacal scales flat, imbricate.

##### Coloration in life

**(Fig. 5).** Ground color of the head, body, limbs, and tail brown; faint, diffuse mottling on the top of the head; thin, dark brown postorbital stripe; ventral portion of lores and suborbital region dark brown; nuchal band faint, bearing two dark-colored posterior projections; paired dark brown paravertebral blotches on nape; three wide faint irregularly shaped body bands edged in dark brown between limb insertions; band interspaces bearing irregularly shaped dark-colored markings; dark-colored speckling on limbs and digits; digits bearing pale-colored bands; eight wide dark-colored caudal bands separated by seven pale-colored bands; first six dark-colored and seven pale-colored caudal bands encircle tail; all ventral surfaces beige, generally immaculate; iris orange-gold in color.

##### Variation

**(Fig. 5).** Individuals of the type series are very similar in overall coloration and pattern. TL and TW of complete original tails (ZMKU R 00959–00960, ZMKU R 00963–00964) are 79.7–97.6 mm (mean 91.4 ± 8.2 mm; *N* = 4) and 4.0–5.2 mm (mean 4.5 ± 0.5; *N* = 4), respectively. The posterior one-third of the tail of ZMKU R 00958 is regenerated (TL 72.0 mm, TW 4.9 mm); that of ZMKU R 00962 is missing (TL 83.8 mm, TW 4.4 mm); and that of ZMKU R 00961 is broken one-third of the way passed the base and nearly all of the broken section is regenerated (TL 73.6 mm, TW 4.07 mm). Regenerated tail sections lack a color pattern. ZMKU R 00963–64 have seven dark-colored and six pale-colored caudal bands as opposed to eight and seven bands, respectively, in the holotype. ZMKU R 00958, ZMKU R 00960, ZMKU R 00962, ZMKU R 00964 are slight less boldly marked than the holotype. Meristic and morphometric differences are listed in Table 5.

##### Distribution.

*Cyrtodactylusfluvicavus* sp. nov. is currently known from the type locality at Tham Than Lot Noi-Tham Than Lot Yai Nature Trail in Chaloem Rattanakosin National Park, Si Sawat District, Kanchanaburi Province, western Thailand (Fig. 1).

##### Etymology.

The specific epithet *fluvicavus* comes from the Latin *fluvius*, meaning stream, river, or flow and the Latin *cavus*, meaning hollow or hole and refers to a landmark cave in the Chaloem Rattanakosin National Park which has a stream that flows through it.

##### Comparisons.

*Cyrtodactylusfluvicavus* sp. nov. is the sister species to a clade composed of ten lineages in a phylogenetic sequence of *C.* sp.9, *C.* sp.10, *Cyrtodactylusuthaiensis* sp. nov., *C.* sp.11, *C.interdigitalis*, C.cf.ngati1, C.cf.ngati2, *C.ngati*3, and the sister lineages *C.ngati*4 and *C.ngati* (Fig. 2). *Cyrtodactylusfluvicavus* sp. nov. differs from those lineages by an uncorrected pairwise sequence divergence of 9.55–12.13% and from all members of the *brevipalmatus* group by 11.61–20.26% (Table 2). It differs categorically from *C.elok* by having as opposed to lacking paravertebral tubercles and femoral pores, and by having 14–18 as opposed to 4–7 longitudinal rows of tubercles. It differs from *C.brevipalmatus*, *C.interdigitalis*, *C.ngati*, *C.ngati*3, *C.rukhadeva*, and *C.* sp.9 in having statistically significant different mean values of the morphometric characters of AG, HumL, ForL, FemL, TibL, HL, HW, HD, EE, ES, EN, EL and IN (Table 6). It differs further from *C.brevipalmatus*, *C.interdigitalis*, *C.ngati*, *C.ngati*3, *C.rukhadeva*, and *C.* sp.9 in having statistically significant different mean values of the meristic characters of SL, PVT, LRT, VS, VSM, TL4E, TL4T, FL4E, FL4U, FL4T, FS, PCS, and BB. Statistically significant and discrete differences between *Cyrtodactylusfluvicavus* sp. nov. and all other species and populations are presented in Tables 4–6.

##### Natural history.

**Figure 6.**
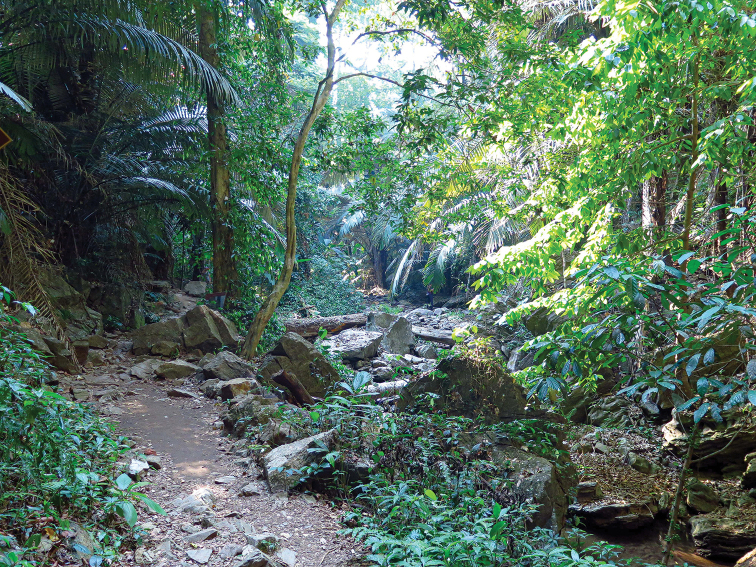
Habitat of *Cyrtodactylusfluvicavus* sp. nov. at Thailand, Kanchanaburi Province, Si Sawat District, Khao Chot Subdistrict, Chaloem Rattanakosin National Park.

All individuals were found in karst forests bearing mixed deciduous and dry evergreen trees amidst rocky streams and a nearby waterfall (Fig. 6). This area is surrounded by agricultural fields and residential areas. Specimens (*N* = 7) were collected at night (1900–2100 h) during the dry season (April) on the tree trunks or palm tree leaves (57.1%; *N* = 4), twigs of shrubs (14.3%; *N* = 1), karst walls (14.3%; *N* = 1), and a wooden bridge (14.3%; *N* = 1) at 526 m elevation with a temperature of 31.9 °C and relative humidity of 56.9%. The holotype (ZMKU R 00959) and two specimens (ZMKU R 00960, ZMKU R 00962) were found on tree trunks ≤ 100 cm above ground level. One specimen (ZMKU R 00964) was found on a palm tree branch approximately 50 cm above the ground. Another specimen (ZMKU R 00963) was found on the twig of a shrub. Another specimen (ZMKU R 00961) was found on a karst wall approximately 3 m above the ground, and another (ZMKU R 00958) on a wooden bridge over a stream. Given these observations, this species appears to be an arboreal habitat generalist. The new species was found to co-occur with two other species of gekkonid lizards, *Cyrtodactylusmonilatus* Yodthong, Rujirawan, Stuart, Grismer, Aksornneam, Termprayoon, Ampai & Aowphol, 2022 and *Dixoniussiamensis* (Boulenger, 1899).

**Table 4.** Summary statistics of the normalized morphometric data for the putative species of the *Cyrtodactylusbrevipalmatus* group. SD = ± standard deviation. Character abbreviations are listed in the Materials and methods.

Species	SVL	AG	HumL	ForL	FemL	TibL	HL	HW	HD	ED	EE	ES	EN	IO	EL	IN
***C.brevipalmatus*** (*N* = 5)																
Mean	1.82	1.49	0.96	0.96	1.06	1.02	1.27	1.10	0.87	0.67	0.75	0.86	0.73	0.68	0.07	0.32
SD	0.020	0.027	0.031	0.023	0.024	0.036	0.012	0.006	0.017	0.041	0.025	0.008	0.019	0.043	0.058	0.041
Lower	1.80	1.46	0.92	0.92	1.02	0.97	1.25	1.10	0.86	0.63	0.71	0.85	0.70	0.62	-0.02	0.25
Upper	1.85	1.52	0.99	0.98	1.09	1.07	1.29	1.11	0.89	0.73	0.77	0.87	0.75	0.73	0.13	0.36
***C.elok*** (*N* = 4)																
Mean	1.91	1.59	0.80	1.05	1.14	1.13	1.34	1.20	1.00	0.71	0.83	0.95	0.79	0.70	0.19	0.43
SD	0.016	0.012	0.341	0.026	0.022	0.024	0.001	0.012	0.008	0.017	0.020	0.008	0.010	0.070	0.062	0.033
Lower	1.89	1.57	0.34	1.02	1.11	1.10	1.34	1.18	0.98	0.68	0.81	0.94	0.78	0.61	0.15	0.40
Upper	1.93	1.60	1.09	1.08	1.16	1.15	1.34	1.21	1.00	0.72	0.85	0.95	0.80	0.76	0.28	0.48
***Cyrtodactylusfluvicavus* sp. nov.** (*N* = 7)																
Mean	1.86	1.52	0.95	1.01	1.11	1.03	1.31	1.14	0.91	0.69	0.78	0.92	0.79	0.73	0.20	0.38
SD	0.027	0.009	0.013	0.017	0.018	0.012	0.007	0.011	0.011	0.007	0.016	0.008	0.011	0.006	0.04	0.02
Lower	1.82	1.51	0.94	0.98	1.10	1.01	1.30	1.13	0.90	0.68	0.76	0.91	0.78	0.72	0.14	0.35
Upper	1.89	1.54	0.98	1.04	1.15	1.05	1.32	1.16	0.93	0.70	0.81	0.94	0.81	0.74	0.24	0.40
***C.interdigitalis*** (*N* = 4)																
Mean	1.86	1.50	0.98	1.01	1.10	1.08	1.30	1.12	0.80	0.72	0.76	0.90	0.77	0.68	0.13	0.33
SD	0.060	0.016	0.032	0.015	0.020	0.007	0.012	0.006	0.187	0.026	0.026	0.024	0.034	0.0307	0.033	0.035
Lower	1.78	1.48	0.94	0.99	1.07	1.07	1.28	1.12	0.56	0.69	0.73	0.88	0.74	0.66	0.10	0.29
Upper	1.91	1.52	1.01	1.02	1.12	1.09	1.31	1.13	0.96	0.75	0.79	0.94	0.81	0.73	0.17	0.38
***Cyrtodactyluskochangensis* sp. nov.** (*N* = 1)																
Value	1.78	1.51	1.00	0.95	1.05	1.02	1.30	1.14	0.91	0.69	0.76	0.91	0.78	0.68	0.05	0.36
**C.cf.kochangensis sp. nov.** (*N* = 1)																
Value	1.85	1.50	1.02	0.94	1.08	1.08	1.27	1.09	0.90	0.72	0.70	0.88	0.75	0.60	0.12	0.36
***C.ngati*** (*N* = 3)																
Mean	1.83	1.47	0.91	0.99	1.06	1.05	1.31	1.08	0.85	0.57	0.76	0.86	0.81	0.74	-0.12	0.43
SD	0.009	0.002	0.004	0.007	0.000	0.006	0.001	0.003	0.010	0.035	0.016	0.015	0.004	0.009	0.019	0.008
Lower	1.82	1.47	0.91	0.98	1.06	1.04	1.31	1.08	0.84	0.55	0.74	0.85	0.80	0.73	-0.13	0.42
Upper	1.84	1.47	0.91	1.00	1.06	1.05	1.31	1.09	0.86	0.61	0.77	0.88	0.81	0.75	-0.09	0.43
***C.ngati*3** (*N* = 3)																
Mean	1.88	1.58	0.94	1.00	1.12	1.07	1.32	1.12	0.95	0.69	0.81	0.93	0.80	0.77	0.08	0.41
SD	0.039	0.001	0.002	0.02	0.001	0.017	0.007	0.002	0.014	0.003	0.004	0.007	0.006	0.022	0.016	0.01
Lower	1.85	1.58	0.93	0.98	1.12	1.05	1.31	1.12	0.94	0.69	0.80	0.92	0.79	0.74	0.07	0.40
Upper	1.92	1.58	0.94	1.02	1.12	1.09	1.33	1.12	0.96	0.69	0.81	0.94	0.80	0.78	0.10	0.42
***C.ngati*4** (*N* = 1)																
Value	1.87	1.50	0.87	1.02	1.13	1.07	1.33	1.11	0.91	0.72	0.79	0.94	0.83	0.75	0.51	0.43
**C.cf.ngati1** (*N* = 1)																
Value	1.89	1.59	0.96	1.03	1.13	1.12	1.34	1.12	0.95	0.85	0.76	0.96	0.82	0.73	0.28	0.49
**C.cf.ngati2** (*N* = 2)																
Mean	1.92	1.59	1.01	1.02	1.17	1.1	1.34	1.15	0.95	0.72	0.80	0.94	0.80	0.65	0.03	0.37
SD	0.035	0.000	0.000	0.000	0.000	0.000	0.000	0.000	0.000	0.000	0.000	0.000	0.000	0.000	0.000	0.000
Lower	1.89	1.59	1.01	1.02	1.17	1.10	1.34	1.15	0.95	0.72	0.80	0.94	0.80	0.65	0.03	0.37
Upper	1.94	1.59	1.01	1.02	1.17	1.10	1.34	1.15	0.95	0.72	0.80	0.94	0.80	0.65	0.03	0.37
***Cyrtodactylusrivularis* sp. nov.** (*N* = 2)																
Mean	1.85	1.53	0.89	0.98	1.04	1.03	1.30	1.16	0.91	0.76	0.80	0.91	0.77	0.75	0.05	0.34
SD	0.025	0.01	0.002	0.007	0.007	0.005	0.005	0.001	0.013	0.007	0.024	0.005	0.004	0.025	0.019	0.016
Lower	1.83	1.53	0.89	0.97	1.03	1.03	1.29	1.16	0.91	0.75	0.79	0.91	0.77	0.74	0.04	0.33
Upper	1.87	1.54	0.90	0.98	1.04	1.04	1.30	1.16	0.92	0.76	0.82	0.91	0.78	0.77	0.06	0.35
**C.rukhadeva and C.cf.rukhadeva** (*N* = 10)																
Mean	1.85	1.49	1.01	0.95	1.05	1.02	1.30	1.14	0.92	0.71	0.75	0.91	0.77	0.67	0.09	0.35
SD	0.026	0.028	0.055	0.029	0.027	0.023	0.009	0.018	0.025	0.036	0.034	0.014	0.015	0.087	0.069	0.022
Lower	1.79	1.45	0.91	0.90	0.99	0.97	1.29	1.10	0.87	0.62	0.70	0.89	0.74	0.46	0.00	0.32
Upper	1.88	1.54	1.08	1.00	1.09	1.05	1.32	1.16	0.95	0.75	0.80	0.92	0.79	0.73	0.23	0.38
***Cyrtodactylusuthaiensis* sp. nov.** (*N* = 1)																
Value	1.76	1.80	0.95	0.99	1.09	1.02	1.28	1.10	0.76	0.72	0.74	0.88	0.75	0.68	0.19	0.32
***C.* sp.9 (Thong Pha Phum**) (*N* = 8)																
Mean	1.86	1.53	0.91	0.97	1.08	1.02	1.30	1.16	0.89	0.71	0.77	0.90	0.77	0.74	0.09	0.34
SD	0.024	0.016	0.034	0.021	0.032	0.019	0.014	0.017	0.008	0.015	0.004	0.012	0.007	0.012	0.061	0.023
Lower	1.81	1.50	0.86	0.93	1.02	0.99	1.27	1.14	0.88	0.69	0.76	0.89	0.76	0.72	0.00	0.30
Upper	1.88	1.55	0.95	1.00	1.12	1.05	1.32	1.19	0.90	0.74	0.78	0.93	0.78	0.76	0.18	0.38
***C.* sp.11** (*N* = 1)																
Value	1.83	1.53	1.01	0.99	1.13	1.09	1.30	1.14	0.75	0.70	0.78	0.92	0.78	0.61	0.08	0.35
***C.* sp.13** (*N* = 2)																
Mean	1.83	1.45	0.94	0.94	1.07	1.01	1.27	1.11	0.91	0.67	0.75	0.88	0.76	0.74	0.15	0.34
SD	0.040	0.000	0.000	0.000	0.000	0.000	0.000	0.000	0.000	0.000	0.000	0.000	0.000	0.000	0.000	0.000
Lower	1.80	1.45	0.94	0.94	1.07	1.01	1.27	1.11	0.91	0.67	0.75	0.88	0.76	0.74	0.15	0.34
Upper	1.86	1.45	0.94	0.94	1.07	1.01	1.27	1.11	0.91	0.67	0.75	0.88	0.76	0.74	0.15	0.34

#### ﻿ Cyrtodactylus rivularis sp. nov.

Taxon classificationAnimaliaSquamataGekkonidae

2BBE9CD2-58DC-5F5B-991D-225EF3E44746

https://zoobank.org/B3381A9D-0049-4C5B-BEB1-DB4D60509F0E

**Figure 7.**
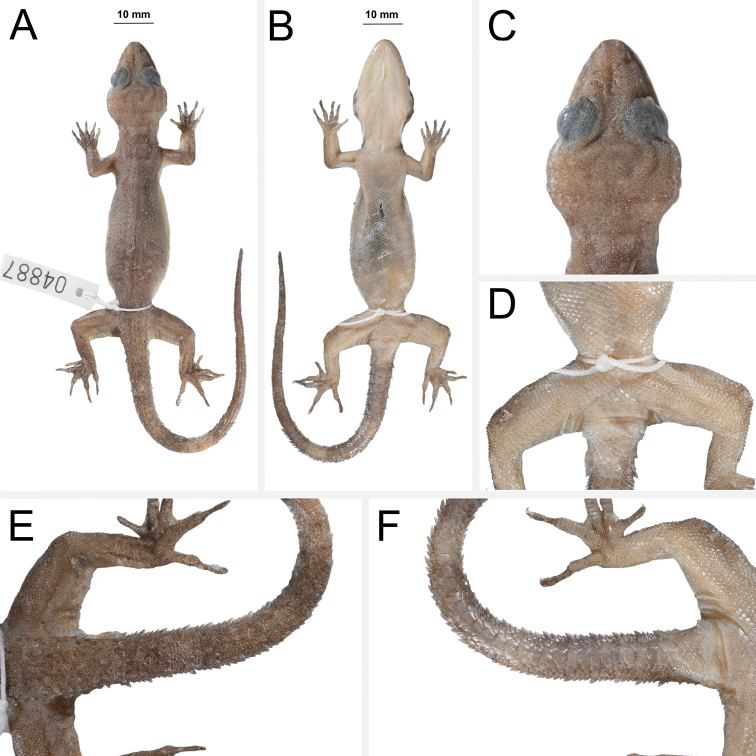
Holotype of *Cyrtodactylusrivularis* sp. nov. ZMKU R 00947 (field no. AA 04887) from Thailand, Prachuap Khiri Khan Province, Hua Hin District, Huai Sat Yai Subdistrict, Kaeng Krachan National Park, Pa La-U Waterfall **A** dorsal view **B** ventral view **C** dorsal view of head **D** ventral view of femoral and precloacal regions **E** dorsal view of tail **F** ventral view of tail.

**Figure 8.**
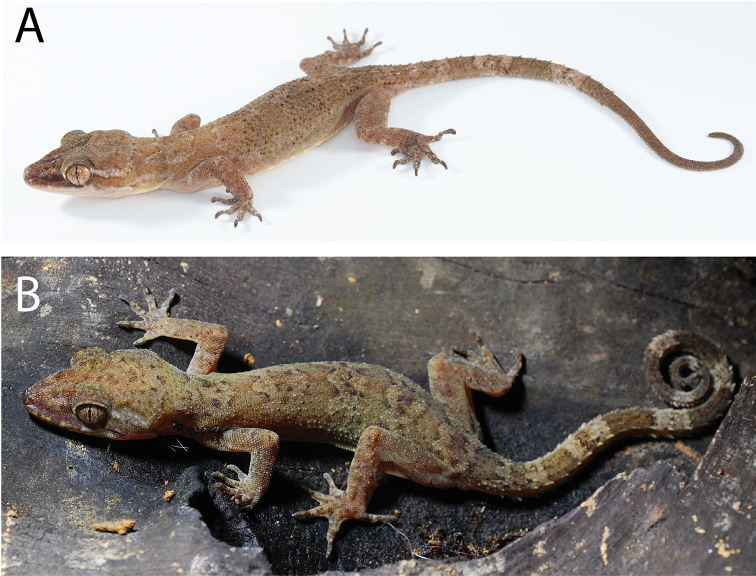
Type series of *Cyrtodactylusrivularis* sp. nov. from Thailand, Prachuap Khiri Khan Province, Hua Hin District, Huai Sat Yai Subdistrict, Kaeng Krachan National Park, Pa La-U Waterfall **A** holotype ZMKU R 00947 **B** paratype ZMKU R 00946.

Figs 7 , 8 Suggested Common Name: Pa La-U Bent-toed Gecko

##### Holotype.

Adult female ZMKU R 00947 from Pa La-U Waterfall, Kaeng Krachan National Park, Huai Sat Yai Subdistrict, Hua Hin District, Prachuap Khiri Khan Province, Thailand (12.53685°N, 99.45972°E, 368 m a.s.l.), collected by Attapol Rujirawan, Siriporn Yodthong, Korkhwan Termprayoon, Natee Ampai, and Piyawan Puanprapai on 15 September 2017.

##### Paratype.

Adult female ZMKU R 00946 bearing the same data as the holotype.

##### Diagnosis.

*Cyrtodactylusrivularis* sp. nov. can be separated from all other species of the *brevipalmatus* group by the combination of having 12 or 13 supralabials, 9–11 infralabials, 33 or 34 paravertebral tubercles, 18–20 rows of longitudinally arranged tubercles, 34–37 transverse rows of ventrals, 160–166 longitudinal rows of ventrals, nine expanded subdigital lamellae on the fourth toe, 12 or 13 unexpanded subdigital lamellae on the fourth toe, 21 or 22 total subdigital lamellae on the fourth toe; eight expanded subdigital lamellae on the fourth finger, 10–12 unexpanded subdigital lamellae on the fourth finger, 18–20 total subdigital lamellae on the fourth finger; 14–16 total enlarged femoral scales; 15 enlarged precloacals; enlarged femorals and enlarged precloacals not continuous, and lacking pores; proximal femorals less than one-half the size of the distal femorals; small tubercles on forelimbs and flanks; large dorsolateral caudal tubercles and wide ventrolateral caudal fringe; ventrolateral caudal fringe composed generally homogeneous scales; tail square in cross-section; single enlarged unpaired medial subcaudals not posteromedially furrowed; maximum SVL 73.9 mm; three or four dark transverse body bands (Tables 4, 5).

##### Description of holotype

**(Fig. 7).** Adult female SVL 73.9 mm; head moderate in length (HL/SVL 0.27), width (HW/HL 0.73), depth (HD/HL 0.40), distinct from neck, triangular in dorsal profile; lores concave slightly anteriorly, weakly inflated posteriorly; prefrontal region concave; canthus rostralis rounded; snout elongate (ES/HL 0.41), rounded in dorsal profile; eye large (ED/HL 0.29); ear opening horizontally elongate, small; eye to ear distance greater than diameter of eye; rostral rectangular, divided dorsally by a deep furrow, bordered posteriorly by large left and right supranasals and one slightly smaller azygous internasal, bordered laterally by first supralabials; external nares bordered anteriorly by rostral, dorsally by large supranasal, posteriorly by two smaller postnasals, bordered ventrally by first supralabial; 13R/12L rectangular supralabials, first two largest, then tapering abruptly below eye; 11R/10L infralabials tapering smoothly to just below eye and then more rapidly beyond posterior margin of eye; scales of rostrum and lores flat to domed, larger than granular scales on top of head and occiput; scales of occiput intermixed with distinct, small tubercles; superciliaries subrectangular, largest anteriorly; mental triangular, bordered laterally by first infralabials, posteriorly by large left and right elongate postmentals contacting medially for approximately 40% of their length posterior to mental; one row of two (R) and four (L) slightly enlarged sublabials extending posteriorly to third(L) and second(R) infralabial, subsequent sublabials much smaller; gular and throat scales small, granular, grading posteriorly into slightly larger, flatter, smooth, imbricate, pectoral and ventral scales.

Body relatively short (AG/SVL 0.47) with well-defined ventrolateral folds; dorsal scales small, granular, interspersed with larger conical, semi-regularly arranged, weakly keeled tubercles; tubercles extend from occipital region onto base of tail and slightly beyond as paravertebral rows; tubercles of nape and occiput small; approximately 20 longitudinal rows of tubercles at midbody; approximately 34 paravertebral tubercles; tubercles on flanks nearly same size as those on dorsum; 34 longitudinal rows of flat, imbricate, ventral scales much larger than dorsal scales; 160 transverse rows of ventral scales; no pore-bearing, precloacal scales; 15 enlarged precloacal scales; no deep precloacal groove or depression; and three rows of post-precloacal scales on midline.

Forelimbs moderate in stature, relatively short (ForL/SVL 0.13); granular scales of forearm slightly larger than those on body, interspersed with tubercles; palmar scales rounded, slightly raised; digits well-developed, relatively short, inflected at basal interphalangeal joints; digits narrower distal to inflections; subdigital lamellae wide, transversely expanded proximal to joint inflections, narrower transverse lamellae distal to joint inflections; claws well-developed, claw base sheathed by a dorsal and ventral scale; 8R/8L expanded and 11R/11L unexpanded lamellae beneath the fourth finger; hind limbs larger and thicker than forelimbs, moderate in length (TibL/SVL 0.15), covered dorsally by granular scales interspersed with moderately sized, conical tubercles dorsally and posteriorly and anteriorly by flat, slightly larger, subimbricate scales; ventral scales of thigh flat, subimbricate, larger than dorsals; subtibial scales flat, imbricate; no pore-bearing femoral scales; 8R/8L enlarged femoral scales; enlarged femoral scales not contiguous with enlarged precloacal scales, terminating distally at knee; proximal femoral scales smaller than distal femoral scales, the former forming an abrupt union with much smaller, rounded, ventral scales of posteroventral margin of thigh; plantar scales flat; digits relatively long, well-developed, inflected at basal interphalangeal joints; 9R/9L wide, transversely expanded subdigital lamellae on fourth toe proximal to joint inflection that extend onto sole, and 13R/13L unexpanded lamellae beneath the fourth toe; and claws well-developed, sheathed by a dorsal and ventral scale at base.

Posterior one-half of tail regenerated, tail long 91.5 mm (TL/SVL 1.24), 4.8 mm in width at base, tapering to a point; nearly square in cross-section; dorsal scales flat, square bearing large tubercles forming a discontinuous dorsolateral longitudinal row; slightly larger, posteriorly directed, semi-spinose tubercles forming large distinct ventrolateral caudal fringe; scales of ventrolateral fringe generally homogeneous; single medial subcaudals enlarged but not paired; subcaudal scales, larger than dorsal caudal scales; base of tail bearing hemipenial swellings; 2R/2L conical postcloacal tubercles at base of hemipenial swellings; and postcloacal scales flat, imbricate.

##### Coloration in life

**(Fig. 8).** Ground color of the head, body, limbs, and tail pale brown; faint, diffuse mottling on rostrum; lores darkly colored; wide, distinct, pale-colored post-orbital stripe; nuchal band faint, bearing two posterior projections; three very faint, wide irregularly shaped body bands edged in slightly darker brown between limb insertions; band interspaces bearing irregularly shaped, faint, dark-colored markings; dark-colored speckling on limbs and digits; digits bearing pale-colored bands; four wide faint dark-colored caudal bands separated by three pale-colored bands on original portion of tail; all caudal bands encircle tail; all ventral surfaces beige, generally immaculate; iris orangish to coppery in color.

##### Variation

**(Fig. 8).** The paratype (ZMKU R 00946) closely approximates the holotype in overall coloration and pattern except that it is more boldly marked. It has four dark-colored body bands as opposed to three and a complete original tail (TL 89.0mm, TW 4.1 mm) bearing eight dark-colored and seven pale-colored bands. The pale-colored postorbital stripe is slightly thinner and less distinct. Meristic and morphometric differences are listed in Table 5.

##### Distribution.

*Cyrtodactylusrivularis* sp. nov. is currently known from the type locality at Pa La-U Waterfall, Kaeng Krachan National Park, Huai Sat Yai Subdistrict, Hua Hin District Prachuap Khiri Khan Province, Thailand.

##### Etymology.

The specific epithet *rivularis* is derived from the Latin *rivus*, meaning stream, brook, or creek refers to rocky brook or stream habitat of the new species.

##### Comparisons.

*Cyrtodactylusrivularis* sp. nov. is the sister species to *C.rukhadeva* (Fig. 2) from which it differs by an uncorrected pairwise sequence divergence of 3.61% (Table 2). It differs from *C.rukhadeva* and C.cf.rukhadeva by having large versus small dorsolateral caudal tubercles and a wide versus narrow ventrolateral fringe. Although no statistical mean differences were recovered between *Cyrtodactylusrivularis* sp. nov. and *C.rukhadeva* which we attribute to the small sample sizes of both species (*N* = 2), they do respectively differ discretely (at this point) in their ranges of SL (12 or 13 vs. 9–11), PVT (33–34 vs. 27–30), VSM (160–166 vs. 152–154), and TL4T (21–22 vs. 18–20) and the morphometric characters of HumL, ForL, FemL, TibL, HD, ED, and IO (Table 5). Discrete differences among *Cyrtodactylusrivularis* sp. nov. and all other species and populations are presented in Tables 4, 5.

##### Natural history.

**Figure 9.**
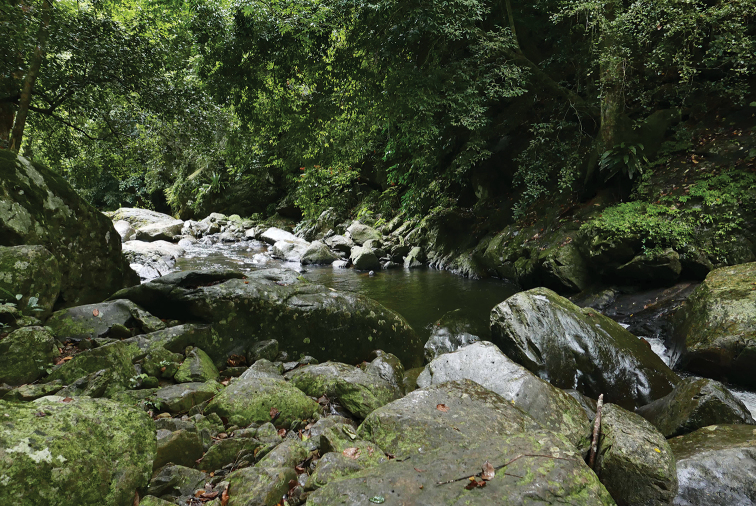
Habitat of *Cyrtodactylusrivularis* sp. nov. at Thailand, Prachuap Khiri Khan Province, Hua Hin District, Huai Sat Yai Subdistrict, Kaeng Krachan National Park, Pa La-U Waterfall.

The holotype and paratype were collected at night (1900–2055 h) on granite boulders by a rocky stream dry evergreen forest at 368 m in elevation (Fig. 9) with a temperature of 25.3 °C and relative humidity of 86.9%. The new species was found to co-occur with two other species of gekkonid lizards, *Cyrtodactylusoldhami* (Theobald, 1876) and *Gehyramutilata* (Wiegmann, 1834).

##### Comments.

*Cyrtodactylusrivularis* sp. nov. and C.cf.rukhadeva occur on separate branches of the phylogeny, thus neither is embedded within one another’s branch indicating there is no evidence of gene flow between them. Both species are reported to occur within the boundaries of Kaeng Krachan National Park (Grismer et al. 2022). Kaeng Krachan National Park is the largest national park in Thailand, encompassing 2,914.7 km^2^ across five districts through the provinces of Phetchaburi and Prachuap Khiri Khan and extending approximately 300 km in latitude. As such, it harbors a wide range of habitats from lowland forests near sea level to cloud forests at its highest peak of 1,513 m in elevation. The seven specimens of C.cf.rukhadeva (THNHM 01807, 24622, 24838, 03251–54) were obtained by various collectors between 11 March 1991 and 25 October 2015 with no specific collection data other than Phetchaburi Province. The locality reported by Grismer et al. (2021c) was an estimate obtained from Ulber (1993) from the central portion of the park in Phetchaburi Province who referred to a specimen he examined (THNHM 24838) as *C.brevipalmatus*. *Cyrtodactylusrivularis* sp. nov. however, is known only from the Park’s southernmost limit in Prachuap Khiri Khan Province. Additional collecting and DNA sequencing will be required to establish the approximate distribution limits of each population.

**Table 5.** Sex and raw meristic, categorical, and morphometric data used in the analyses from specimens in the *Cyrtodactylusbrevipalmatus* group. m = male; f = female; R/L = right/left; / = data unavailable.

Species	*C.brevipalmatus*	C.cf.brevipalmatus	C.cf.brevipalmatus	*C.brevipalmatus*	*C.brevipalmatus*	*C.elok*	*C.elok*	*C.elok*	*C.elok*	*C.interdigitalis*	*C.interdigitalis*	*C.interdigitalis*	*C.interdigitalis*	*C.* sp.11	*C.ngati*	*C.ngati*	*C.ngati*	*C.ngati*	*C.ngati*3	*C.ngati*3	*C.ngati*3	*C.ngati*4	C.cf.ngati1	C.cf.ngati2	C.cf.ngati2	*C.rukhadeva*	*C.rukhadeva*	*C.rukhadeva*	C.cf.rukhadeva	C.cf.rukhadeva	C.cf.rukhadeva	C.cf.rukhadeva	C.cf.rukhadeva	C.cf.rukhadeva	C.cf.rukhadeva	*Cyrtodactylusfluvicavus* sp. nov.	*Cyrtodactylusfluvicavus* sp. nov.	*Cyrtodactylusfluvicavus* sp. nov.	*Cyrtodactylusfluvicavus* sp. nov.	*Cyrtodactylusfluvicavus* sp. nov.	*Cyrtodactylusfluvicavus* sp. nov.	*Cyrtodactylusfluvicavus* sp. nov.	*Cyrtodactyluskochangensis* sp. nov	C.cf.kochangensis	*Cyrtodactylusuthaiensis* sp. nov.	*Cyrtodactylusrivularis* sp. nov.	*Cyrtodactylusrivularis* sp. nov.	*C.* sp.9 Thong Pha Phum	*C.* sp.9 Thong Pha Phum	*C.* sp.9 Thong Pha Phum	*C.* sp.9 Thong Pha Phum	*C.* sp.9 Thong Pha Phum	*C.* sp.9 Thong Pha Phum	*C.* sp.9 Thong Pha Phum	*C.* sp.9 Thong Pha Phum	*C.* sp.13	*C.* sp.13
Institutional catalog number	LSUHC 1899	LSUHC 15076	LSUHC 11788	THNHM 10670	THNHM 14112	LSUHC 8238	LSUHC 12180	LSUHC 12181	ZMMU R-16144	THNHM 20226 paratype	THNHM 20228 paratype	THNHM 20229 paratype	THNHM 20227 paratype	ZMMU R-16492	HNUE-R00111 holotype	IEBR 4829 paratype	VNUF R.2020.12 paratype	HNUE-R00112 paratype	FMNH 255454	FMNH 270493	FMNH 270492	FMNH 265806	NCSM 79472	ZMMU R-14917	NCSM 80100	ZMMU R-16851 holotype	ZMMU R-16852 paratype	ZMKU R 00948 topotype	THNHM 24622	THNHM 24838	THNHM 03251	THNHM 03252	THNHM 03253	THNHM 03254	THNHM 01807	ZMKU R 00959 holotype	ZMKU R 00958 paratype	ZMKU R 00960 paratype	ZMKU R 00961 paratype	ZMKU R 00962 paratype	ZMKU R 00963 paratype	ZMKU R 00964 paratype	ZMKU R 00945 holotype	THNHM 01667	ZMKU R 00949 holotype	ZMKU R 00947 holotype	ZMKU R 00946 paratype	ZMKU R 00950	ZMKU R 00951	ZMKU R 00952	ZMKU R 00953	ZMKU R 00954	ZMKU R 00955	ZMKU R 00956	ZMKU R 00957	THNHM 00104	THNHM 27821
Sex	m	f	f	f	f	f	m	m	f	f	f	f	f	m	m	f	f	f	f	m	m	m	f	f	f	m	f	f	m	f	m	m	f	m	m	m	m	m	f	f	f	f	f	m	m	f	f	f	m	f	m	m	f	m	f	f	f
**Meristic data**																																																									
Supralabials (SL)	11	12	10	14	12	11	8	13	9	14	12	11	12	11	10	10	10	10	13	13	13	10	14	9	12	11	9	14	11	13	13	11	12	13	12	12R/12L	13R/12L	13R/12L	11R/12L	12R/12L	13R/12L	12R/11L	12R/13L	12	13R/15L	13R/12L	13R/12L	12	13	13	14	13	13	13	13	12	15
Infralabials (IL)	8	10	9	11	11	11	8	11	9	9	8	8	7	9	9	9	9	9	10	9	11	8	11	10	12	10	11	9	10	10	10	10	10	11	10	10R/10L	10R/10L	9R/10L	10R/10L	10R/10L	10R/10L	10R/10L	9R/9L	10	10R/11L	11R/10L	10R/9L	8	8	10	10	9	10	10	9	10	11
Paravertebral tubercles (PVT)	39	37	38	37	37	0	0	0	0	32	33	33	33	30	39	40	38	40	28	27	26	27	28	32	29	27	30	30	26	28	27	27	30	30	26	30	28	27	27	28	26	28	34	29	33	34	33	32	33	34	34	36	36	30	30	33	29
Longitudinal rows of tubercles (LRT)	15	16	17	16	14	6	7	4	4	19	20	19	19	18	18	18	17	22	19	18	17	19	18	24	19	19	20	19	18	19	18	18	19	19	19	17	17	14	16	17	18	16	14	19	17	20	18	21	19	20	20	21	21	19	19	18	20
Ventral scales (VS)	38	38	38	36	39	45	45	47	36	42	40	42	43	34	38	36	35	32	37	36	36	33	33	36	35	34	43	38	38	36	37	37	39	34	35	34	37	33	30	36	37	39	35	34	36	34	37	34	33	33	34	30	33	32	33	37	36
Ventral scales along middle of the body (VSM)	176	170	182	154	160	190	225	234	192	187	170	187	178	160	168	164	178	158	159	166	156	158	164	166	165	154	152	165	162	158	157	159	168	160	161	155	154	155	172	164	175	170	172	159	159	160	166	173	158	156	166	159	159	150	169	159	165
Expanded subdigital lamellae on 4^th^ toe (TL4E)	7	8	9	8	8	10	9	9	9	12	10	10	11	9	8	10	9	9	10	10	8	10	9	8	10	9	9	9	8	9	9	10	9	10	10	9R/9L	10R/10L	9R/9L	9R/9L	10R/11L	9R/10L	9R/9L	9R/8L	8	8R/(broken)L	9R/9L	9R/9L	9	10	9	8	10	8	9	9	9	7
Unmodified subdigital lamellae on 4^th^ toe (TL4U)	13	11	11	11	12	11	10	11	9	14	13	12	14	10	11	10	11	10	11	11	11	11	12	10	10	11	11	12	11	13	12	12	15	13	13	11R/11L	12R/11L	10R/10L	12R/12L	11R/11L	10R/10L	12R/13L	12R/11L	13	12R/(broken)L	13R/13L	12R/13L	12	14	13	12	13	12	11	13	12	12
Total subdigital lamellae 4^th^ toe (TL4T)	20	19	20	19	20	21	19	20	18	26	23	22	23	19	13	16	17	16	21	21	19	21	21	18	20	20	18	21	19	22	21	22	14	23	23	20R/20L	22R/21L	19R/19L	21R/21L	21R/22L	19R/20L	22R/22L	21R/19L	21	20	22R/22L	21R/22L	21	24	22	20	23	20	20	22	21	19
Expanded subdigital lamellae on 4^th^ finger (FL4E)	8	8	8	7	8	8	9	9	9	9	8	9	9	10	6	6	7	6	8	8	8	8	9	7	9	9	8	8	7	8	8	8	8	8	8	8R/8L	8R/8L	8R/8L	8R/8L	7R/7L	8R/9L	7R/7L	8R/8L	8	7R/7L	8R/8L	8R/8L	8	7	7	8	8	8	8	8	8	8
Unmodified subdigital lamellae on 4^th^ finger (FL4U)	9	11	10	10	10	12	13	9	8	12	11	12	13	9	9	9	9	9	10	10	10	10	8	9	10	10	9	11	10	11	10	10	12	12	12	10R/10L	10R/10L	10R/9L	11R/11L	10R/10L	9R/9L	10R/10L	10R/10L	12	11R/11L	11R/10L	12R/12L	10	12	12	11	12	12	11	12	11	10
Total subdigital lamellae 4^th^ finger (FL4T)	17	19	18	17	18	20	22	18	17	21	21	21	22	19	15	15	18	15	18	18	18	18	17	16	19	19	17	19	17	17	18	18	20	20	20	18R/18L	18R/18L	18R/17L	19R/19L	17R/17L	17R/18L	17R/17L	18R/18L	20	18R/18L	19R/18L	20R/20L	18	19	19	19	20	20	19	20	19	18
Enlarged femoral scales (R/L)	0	0	0	8R/8L	7R/7L	0	0	0	0	11R/8L	10R/9L	8R/8L	9R/10L	9R/8L	10R/10L	9R/8L	10R/9L	8R/9L	9R/7L	8R/9L	9R/9L	8R/8L	9R/8L	7R/8L	7R/8L	9R/8L	8R/8L	9R/8L	9R/L	9R/9L	9R/7L	7R/7L	6R/7L	5R/8L	7R/7L	5R/6L	4R/5L	5R/6L	6R/6L	5R/6L	5R/6L	6R/6L	6R/6L	7R/7L	8R/8L	8R/8L	6R/8L	5R/7L	8R/8L	8R/8L	7R/8L	8R/8L	7R/8L	7R/6L	8R/8L	9R/9L	7R/10L
Total enlarged femoral scales (FS)	16	10	11	16	14	0	0	0	0	14	19	19	19	17	20	17	19	17	16	17	18	16	17	15	15	17	16	17	18	18	16	14	13	13	14	11	9	11	12	11	11	12	12	14	16	16	14	12	16	16	15	16	15	13	16	18	17
Total femoral pores in males (FP)	7	0	0	0	0	0	0	0	0	0	0	0	0	17	14	0	0	0	0	14	15	13	0	0	0	17	0	0	14	0	12	13	0	11	13	11	8	10	0	0	0	0	0	14	12	0	0	0	16	0	14	15	14	12	0	0	0
Enlarged precloacal scales (PCS)	7	7	7	8	7	8	8	8	7	14	15	13	19	13	13	13	13	13	15	13	13	13	12	13	13	17	13	15	15	15	14	13	15	15	14	15	14	14	15	14	15	15	12	16	14	15	15	17	15	15	15	15	15	15	15	14	16
Precloacal pores in males (PP)	7	0	0	0	0	0	8	8	0	0	0	0	0	13	0	0	0	0	13	13	13	13	0	0	0	17	13	0	15	0	14	13	0	15	14	15	14	14	0	0	0	0	0	16	14	0	0	0	15	0	15	15	15	15	0	0	0
Postcloacal tubercles (PCT)	3	3	2	3	3	3	2	3	3	3	2	3	3	3	3	2	1	2	0	0	0	0	2	3	4	3	2	2R/3L	3	2	3	2	2	3	2	3R/2L	3R/2L	3R/3L	1R/1L	3R/2L	3R/3L	2R/2L	1R/1L	3	3R/3L	2R/2L	3R/3L	2	2R/3L	3	3	2R/3L	2R/3L	3	2	3	3
Body bands (BB)	4	6	3	5	5	5	5	3	3	5	5	5	5	3	6	6	6	6	3	4	3	3	3	3	3	3	3	3	3	3	4	4	/	/	5	3	3	3	3	3	3	3	5	5	6	3	4	3	4	3	4	3	5	4	4	3	/
**Categorical data**																																																									
Small tubercles on flank (FKT)	present	present	present	present	present	absent	absent	absent	absent	present	present	present	present	present	present	present	present	present	present	present	present	present	present	present	present	present	present	present	present	present	present	present	present	present	present	present	present	present	present	present	present	present	present	present	present	present	present	present	present	present	present	present	present	present	present	present	present
Dorsolateral caudal tubercles (DCT)	small	small	small	/	small	large	large	large	large	small	/	small	small	large	small	small	small	small	small	small	small	small	small	small	small	small	small	small	small	small	small	small	small	small	/	small	small	small	small	small	small	small	large	large	large	large	large	large	large	large	large	large	large	/	large	small	small
Ventrolateral caudal fringe narrow or wide (VLF1)	narrow	narrow	narrow	/	narrow	wide	wide	wide	wide	narrow	/	narrow	narrow	wide	narrow	narrow	narrow	narrow	narrow	narrow	narrow	narrow	narrow	narrow	narrow	narrow	narrow	narrow	narrow	narrow	narrow	narrow	narrow	narrow	/	narrow	narrow	narrow	narrow	narrow	narrow	narrow	wide	wide	wide	wide	wide	wide	wide	wide	wide	wide	wide	/	wide	narrow	narrow
Ventrolateral caudal fringe scales generally homogenous (VLF2)	no	no	no	/	no	no	no	no	no	yes	yes	yes	yes	yes	no	no	no	no	yes	yes	yes	yes	yes	yes	yes	yes	yes	yes	yes	yes	yes	yes	yes	yes	/	no	no	no	no	no	no	no	no	no	no	yes	yes	no	no	no	no	no	no	/	no	yes	yes
Tail cross-section (TLcross)	circular	circular	circular	/	circular	square	square	square	square	circular	/	circular	circular	square	circular	circular	circular	circular	circular	circular	circular	circular	circular	circular	circular	square	square	square	square	square	square	square	square	square	/	circular	circular	circular	circular	circular	circular	circular	square	/	circular	square	square	square	square	square	square	square	square	/	square	circular	circular
Slightly enlarged medial subcaudals (SC1)	present	present	present	/	absent	absent	absent	absent	absent	absent	/	absent	absent	present	present	present	present	present	/	present	present	present	present	present	present	absent	absent	absent	absent	absent	absent	absent	absent	absent	/	present	present	present	present	present	present	present	present	present	present	absent	absent	present	present	present	present	present	present	/	present	present	present
Single enlarged medial subcaudal (SC2)	absent	absent	absent	/	absent	absent	absent	absent	absent	absent	/	absent	absent	absent	absent	absent	absent	absent	/	absent	absent	absent	absent	absent	absent	present	present	present	present	present	present	present	present	present	/	absent	absent	absent	absent	absent	absent	absent	absent	absent	absent	present	present	absent	absent	absent	absent	absent	absent	/	absent	absent	absent
Enlarged medial subcaudals intermittent, medially furrowed, posteriorly emarginate (SC3)	no	no	no	/	no	no	no	no	no	yes	/	yes	yes	no	no	no	no	no	/	no	no	no	no	no	no	no	no	no	no	no	no	no	no	no	no	no	no	no	no	no	no	no	no	no	yes	no	no	no	no	no	no	yes	no	/	no	no	no
**Morphometric data**																																																									
SVL	68.8	70.8	64.1	65.95	63.79	80.2	78.2	84.8	78.6	81.19	74.80	78.56	59.70	68.1	66.5	68.1	69.3	46.6	83.6	70.2	74.1	73.8	78.0	87.1	77.7	74.9	71.7	71.6	68.3	71.8	73.6	75.3	74.7	73.2	61.5	72.5	72.0	69.6	68.4	76.8	65.7	78.2	60.1	70.2	58.1	73.9	68.1	73.1	73.5	73.7	73.2	64.4	76.6	76.6	74.2	63.7	72.9
AG	35.7	33.4	30.1	30.0	26.5	39.7	37.8	41.5	36.2	34.5	33.7	32.7	24.6	34.6	28.8	29.8	30.2	19.7	41.3	35.4	37.0	31.3	38.2	41.9	36.8	34.6	32.6	33.9	27.3	29.9	30.9	31.3	32.2	30.3	26.2	33.4	33.6	32.0	30.4	35.6	30.6	38.1	29.0	31.5	26.6	34.8	33.2	34.8	33.9	35.4	33.6	28.5	37.1	33.2	35.1	25.8	30.6
HumL	9.7	9.3	8.0	9.6	9.5	10.2	9.1	10.1	1.7	9.8	10.2	11.2	7.4	10.3	7.9	8.1	8.5	5.6	8.6	8.7	8.6	6.9	8.7	11.5	9.2	10.7	10.4	7.9	9.8	8.3	12.2	11.3	11.8	11.0	10.1	9.1	8.8	9.0	8.0	10.0	7.5	10.1	6.5	10.2	7.0	8.1	7.6	8.4	7.2	9.0	9.0	7.2	8.0	8.1	8.6	7.6	10.1
ForL	9.9	9.8	8.9	8.2	8.7	11.5	11.7	11.8	10.2	10.6	10.5	11.1	8.4	8.5	9.2	10.0	10.1	6.5	10.2	9.3	10.4	10.0	10.3	10.4	10.7	8.6	7.9	9.6	8.7	8.5	9.0	10.6	9.6	9.2	7.9	10.5	10.3	10.5	10.1	11.1	8.8	10.8	7.6	8.6	8.3	9.7	9.1	9.5	9.1	9.2	9.8	9.2	10.0	8.6	9.8	8.1	9.6
FemL	12.0	12.6	11.5	11.7	9.8	12.9	14.2	14.6	13.1	14.7	13.2	12.7	10.2	12.6	11.5	11.5	11.5	7.6	13.7	12.7	13.0	13.1	13.1	15.2	14.2	12.6	11.8	10.5	10.8	10.9	11.5	10.2	11.9	12.1	9.5	13.1	12.5	12.5	13.5	14.1	11.5	13.9	10.4	12.1	10.0	11.4	10.4	12.8	11.6	12.3	12.5	10.9	13.7	10.8	12.5	10.7	12.8
TibL	11.6	12.2	10.5	9.7	8.2	13.5	14.0	13.8	12.3	13.1	11.9	12.9	10.2	11.4	10.8	11.1	11.8	7.8	12.5	11.8	11.2	11.1	12.8	12.6	12.7	10.1	9.3	11.2	9.7	10.7	10.9	11.7	11.3	11.1	9.1	11.3	10.6	10.2	9.9	11.2	9.4	12.3	8.4	11.8	8.4	11.2	10.3	10.5	10.1	10.6	10.6	9.9	11.1	10.0	11.4	10.1	10.2
HL	19.3	19.3	19.0	17.9	18.2	21.8	21.6	21.9	21.7	20.8	19.9	21.7	16.7	18.4	20.1	20.4	20.7	16.1	21.7	20.6	20.3	20.7	21.2	22.1	21.4	20.2	19.2	19.7	19.7	19.9	20.8	21.3	20.8	21.5	17.9	20.1	20.5	19.7	20.1	21.2	18.6	21.3	17.3	18.3	16.1	20.3	19.3	19.9	20.9	20.1	20.0	17.6	20.4	19.3	20.0	17.6	19.9
HW	13.2	13.8	12.3	12.3	12.0	15.6	16.1	15.9	15.1	14.0	13.4	14.2	11.4	13.1	12.6	12.0	11.8	8.8	13.8	12.5	13.0	12.3	12.7	14.8	13.5	14.6	13.4	14.0	13.1	13.9	14.9	15.0	13.1	14.1	11.8	14.0	13.4	12.9	13.0	14.9	13.0	15.4	11.6	12.1	10.9	14.9	13.7	14.5	14.3	15.7	13.9	12.8	14.7	14.4	14.1	11.9	13.8
HD	8.0	7.6	7.6	7.3	7.0	9.6	9.8	10.4	9.8	3.4	8.6	8.7	6.6	8.3	7.4	7.2	6.6	5.1	9.2	8.4	9.1	7.6	8.3	8.7	9.2	9.2	8.5	8.3	7.3	8.9	8.2	8.2	8.1	8.9	7.5	8.5	8.1	8.3	7.9	8.1	7.8	8.3	6.5	7.8	6.3	8.2	8.2	7.8	7.7	7.9	7.7	7.0	8.2	7.8	7.6	7.7	8.4
ED	5.2	4.5	4.3	5.3	4.4	4.8	5.0	5.7	5.0	5.3	5.5	5.9	4.4	4.4	3.8	4.1	3.4	2.6	4.9	4.9	4.9	4.8	6.5	4.6	6.0	4.6	4.3	5.5	4.9	5.1	5.8	5.4	5.0	5.5	4.7	5.0	5.0	4.9	4.7	5.1	4.5	5.3	4.2	5.2	4.6	5.8	5.6	5.0	5.1	5.0	5.0	4.8	5.6	5.3	4.9	4.1	5.3
EE	5.7	5.9	4.9	5.7	5.7	6.4	7.1	7.0	6.8	5.8	6.2	6.4	4.8	6.2	5.8	5.5	5.9	4.4	6.9	6.1	6.2	5.7	5.3	6.5	6.2	6.2	6.2	5.8	5.1	6.2	5.6	5.7	5.4	6.2	4.3	6.5	5.9	5.7	5.8	6.1	5.4	6.5	5.0	4.9	4.7	6.5	6.2	5.9	5.9	6.0	5.9	5.3	6.1	6.0	6.0	4.9	6.3
ES	7.4	7.6	7.0	7.0	7.2	8.6	8.7	9.5	8.6	8.3	7.8	9.1	6.8	7.7	7.5	7.6	6.9	5.0	9.0	8.3	8.3	8.2	8.7	8.8	8.4	8.3	7.7	7.9	7.4	8.1	8.4	8.8	8.1	8.6	7.3	8.5	8.3	8.2	8.1	9.2	7.3	9.3	6.9	7.5	6.4	8.3	7.9	7.9	8.5	7.9	7.9	7.3	8.2	7.9	7.9	7.2	8.0
EN	5.7	5.4	4.9	5.3	5.4	6.0	6.2	6.5	6.2	6.0	5.5	6.8	5.1	5.5	6.7	6.3	6.2	4.5	6.5	6.2	6.1	6.2	6.2	6.6	6.0	6.3	5.7	5.8	5.4	6.0	6.2	6.4	5.8	6.2	5.3	6.5	6.2	5.9	6.1	6.6	5.6	6.5	5.2	5.5	4.9	6.1	5.8	6.0	6.1	6.0	5.8	5.4	6.1	6.0	5.9	5.6	5.9
IO	5.4	4.7	4.7	4.2	5.2	5.7	5.4	5.4	3.9	4.8	4.7	5.5	4.3	2.9	5.6	5.4	5.6	4.2	6.6	5.6	5.4	5.1	4.9	3.5	5.7	3.3	3.1	5.6	4.5	4.7	5.6	5.7	5.7	5.6	4.2	5.5	5.4	5.3	5.1	5.6	5.0	5.6	4.2	4.0	4.3	5.8	5.5	5.4	5.5	5.8	5.5	4.9	5.7	5.6	5.3	4.8	6.1
EL	1.0	1.4	1.1	1.3	1.0	1.9	1.4	1.5	1.4	1.3	1.3	1.6	1.2	0.9	0.8	0.8	0.7	0.3	1.3	1.1	1.2	1.0	1.5	1.2	0.9	1.2	1.0	1.4	1.6	1.5	1.2	1.3	1.2	1.2	0.9	1.4	1.5	1.7	1.4	1.8	1.6	1.8	1.0	1.3	1.5	1.1	1.1	1.1	1.5	1.5	1.2	1.2	1.0	1.2	1.3	1.4	1.4
IN	1.7	2.1	2.3	2.1	2.2	2.7	2.6	2.5	3.1	2.1	2.2	2.5	1.8	2.3	2.8	2.6	2.6	2.0	2.8	2.5	2.5	2.3	2.7	2.2	2.5	2.2	2.1	2.1	2.0	2.2	2.4	2.5	2.4	2.3	2.0	2.3	2.4	2.5	2.3	2.3	2.3	2.6	1.9	2.2	1.8	2.3	2.0	2.3	2.4	2.2	2.0	2.0	2.3	2.2	2.2	2.1	2.3

#### ﻿ Cyrtodactylus kochangensis sp. nov.

Taxon classificationAnimaliaSquamataGekkonidae

7E67190E-06B0-564C-B4C8-E5A323705A73

https://zoobank.org/96DF655B-BBB7-4C06-9418-744EBCD14703

**Figure 10.**
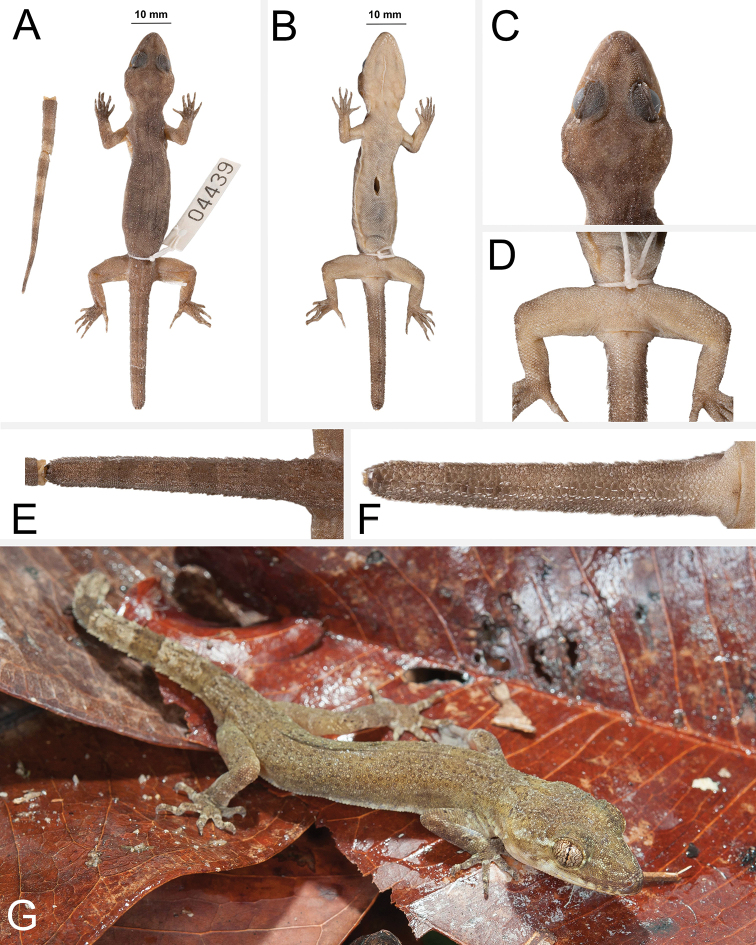
Holotype of *Cyrtodactyluskochangensis* sp. nov. ZMKU R 00945 (field no. AA 04439) from Thailand, Ranong Province, Mueng Ranong District, Ko Phayam Subdistrict, Ko Chang **A** dorsal view **B** ventral view **C** dorsal view of head **D** ventral view of femoral and precloacal regions **E** dorsal view of tail **F** ventral view of tail **G** holotype in life.

Fig. 10 Suggested Common Name: Ko Chang Bent-toed Gecko

##### Holotype.

Adult female ZMKU R 00945 from Ko Chang Island, Ko Phayam Subdistrict, Mueang Ranong District, Ranong Province, Thailand (9.82411°N, 98.43999°E, 36 m a.s.l.), collected by Siriporn Yodthong, Natee Ampai, Attapol Rujirawan, and Piyawan Puanprapai on 8 July 2017.

##### Additional material examined.

Cyrtodactyluscf.kochangensis sp. nov. adult male THNHM 01667 from Khlong Naka Wildlife Sanctuary, Suk Samran District, Ranong Province, Thailand (~N 9.4596, E 98.5044, elevation unknown), collected by Yodchaiy Chuaynkern between 28 December 2000 and 2 January 2001.

##### Diagnosis

**(based on the holotype).***Cyrtodactyluskochangensis* sp. nov. can be separated from all other species of the *brevipalmatus* group by the combination of having 12 or 13 supralabials, nine infralabials, 34 paravertebral tubercles, 14 rows of longitudinally arranged tubercles, 35 transverse rows of ventrals, 172 longitudinal rows of ventrals, 8 or 9 expanded subdigital lamellae on the fourth toe, 11 or 12 unexpanded subdigital lamellae on the fourth toe, 19–21 total subdigital lamellae on the fourth toe; eight expanded subdigital lamellae on the fourth finger, ten unexpanded subdigital lamellae on the fourth finger, 18 total subdigital lamellae on the fourth finger; 12 total enlarged femoral scales; 12 enlarged precloacal scales; enlarged femoral and enlarged precloacal scales not continuous; proximal femoral scales less than one-half the size of the distal femorals; small tubercles on forelimbs and flanks; large dorsolateral caudal tubercles and a wide ventrolateral caudal fringe not composed homogeneous scales; tail square in cross-section; slightly enlarged paired medial subcaudals not posteromedially furrowed; maximum SVL 60.1 mm; five dark transverse body bands (Tables 4, 5).

##### Description of holotype

**(Fig. 10).** Adult female SVL 60.1 mm; head moderate in length (HL/SVL 0.29), width (HW/HL 0.67), depth (HD/HL 0.38), distinct from neck, triangular in dorsal profile; lores flat anteriorly, weakly inflated posteriorly; prefrontal region slightly concave; canthus rostralis rounded; snout elongate (ES/HL 0.40), rounded in dorsal profile; eye large (ED/HL 0.24); ear opening subcircular, small; eye to ear distance greater than diameter of eye; rostral rectangular, furrowed dorsally, bordered posteriorly by large left and right supranasals and one slightly smaller azygous internasal, bordered laterally by first supralabials; external nares bordered anteriorly by rostral, dorsally by large supranasal, posteriorly by two slightly smaller postnasals, bordered ventrally by first supralabial; 12R/13L rectangular supralabials tapering smoothly to below eye; 9R/9L infralabials tapering smoothly to below eye; scales of rostrum and lores domed, slightly larger than granular scales on top of head and occiput; scales of occiput intermixed with distinct, small tubercles; superciliaries subrectangular, largest anteriorly; mental triangular, bordered laterally by first infralabials and posteriorly by large left and right trapazoidal postmentals contacting medially for approximately 40% of their length posterior to mental; one row of six (R,L) slightly enlarged sublabials extending posteriorly fifth infralabials, subsequent sublabials much smaller; gular and throat scales small, granular, grading posteriorly into slightly larger, flatter, smooth, imbricate, pectoral and ventral scales.

Body relatively short (AG/SVL 0.48) with well-defined ventrolateral folds; dorsal scales small, granular, interspersed with larger conical, semi-regularly arranged, weakly keeled tubercles; tubercles extend from occipital region onto base of tail and slightly beyond as paravertebral rows; tubercles of nape and occiput smaller than those on body; approximately 14 longitudinal rows of tubercles at midbody; approximately 34 paravertebral tubercles; tubercles on upper flanks smaller than those on dorsum; approximately 35 longitudinal rows of flat, imbricate, ventral scales much larger than dorsal scales; approximately 172 transverse rows of ventral scales; no pore-bearing, precloacal scales; 12 enlarged precloacal scales; no deep precloacal groove or depression; and approximately three rows of post-precloacal scales on midline.

Forelimbs moderate in stature, relatively short (ForL/SVL 0.13); granular scales of forearm slightly larger than those on body, interspersed with tubercles; palmar scales rounded, slightly raised; digits well-developed, relatively short, inflected at basal interphalangeal joints; digits narrower distal to inflections; subdigital lamellae wide, transversely expanded proximal to joint inflections, narrower transverse lamellae distal to joint inflections; claws well-developed, claw base sheathed by a dorsal and ventral scale; 8R/8L expanded and 10R/10L unexpanded lamellae beneath the fourth finger; hind limbs larger and thicker than forelimbs, moderate in length (TibL/SVL 0.14), covered dorsally by granular scales interspersed with moderately sized, conical tubercles dorsally and posteriorly and anteriorly by flat, slightly larger, subimbricate scales; ventral scales of thigh flat, subimbricate, larger than dorsals; subtibial scales flat, imbricate; no pore-bearing femoral scales; 6R/6L enlarged femoral scales; enlarged femoral scales not contiguous with enlarged precloacal scales, terminating distally at knee; proximal femoral scales smaller than distal femorals, the former forming an abrupt union with much smaller, rounded, ventral scales of posteroventral margin of thigh; plantar scales flat; digits relatively long, well-developed, inflected at basal interphalangeal joints; 9R/8L wide, transversely expanded subdigital lamellae on the fourth toe proximal to joint inflection that extend onto sole, and 12R/11L unexpanded lamellae beneath the fourth toe; and claws well-developed, sheathed by a dorsal and ventral scale at base.

Tail original (but in two pieces), long 80.3 mm (TL/SVL 1.34), 4.0 mm in width at base, tapering to a point; nearly square in cross-section; dorsal scales flat, square bearing tubercles forming paravertebral rows and large tubercles forming a dorsolateral longitudinal row; slightly larger, posteriorly directed, semi-spinose tubercles forming wide distinct ventrolateral caudal fringe; scales of ventrolateral fringe generally interspersed at regular intervals with larger spinose scales; medial subcaudal scales paired, slightly enlarged; subcaudals, larger than dorsal caudal scales; base of tail bearing hemipenial swellings; one conical postcloacal tubercle at base of hemipenial swellings; and postcloacal scales flat, imbricate.

##### Coloration in life

**(Fig. 10).** Ground color of the head, body, limbs, and tail pale brown; faint, diffuse mottling on rostrum; lores darkly colored; wide, distinct, pale-colored postorbital stripe; nuchal band faint, bearing two posterior projections; three very faint, wide, irregularly shaped body bands between limb insertions edged in slightly darker brown; band interspaces bearing irregularly shaped, faint, dark-colored markings; dark-colored speckling on limbs and digits; digits bearing pale-colored bands; four wide, faint, dark-colored caudal bands separated by three pale-colored bands on original portion of tail; all caudal bands encircle tail; all ventral surfaces beige, generally immaculate; and iris orangish to coppery in color.

##### Variation.

The additional specimen (THNHM 01667) closely approximates the holotype in overall coloration and pattern except that it is more boldly marked. It has four dark-colored body bands as opposed to three and a complete original tail bearing eight dark-colored and seven pale-colored bands. The pale-colored postorbital stripe is slightly thinner and less distinct. Meristic and morphometric differences are listed in Table 5. Given its overall morphological and color pattern similarities and close geographic proximity to the holotype (~ 40 km), we consider this individual as C.cf.kochangensis sp. nov. pending genetic data.

##### Distribution.

*Cyrtodactyluskochangensis* sp. nov. is currently known only from the type locality at Ko Chang Island, Ko Phayam Subdistrict, Mueang Ranong District, Ranong Province, Thailand. The additional population of pending species status occurs in the Khlong Naka Wildlife Sanctuary, Suk Samran District Ranong Province.

##### Etymology.

The specific epithet *kochangensis* is in reference to the type locality, Ko Chang, Ranong Province, Thailand

##### Comparisons

**(based on the holotype)**. *Cyrtodactyluskochangensis* sp. nov. forms a clade with the sister species *Cyrtodactylusrivularis* sp. nov. and *C.rukhadeva* (Fig. 2) from which it differs by an uncorrected pairwise sequence divergence of 12.00–12.52% and 12.52–13.68%, respectively (Table 2) and it and C.cf.kochangensis sp. nov. are separated from *C.rukhadeva* and *Cyrtodactylusrivularis* sp. nov. by geographic distance of no less than ~280–470 km (Fig. 1). The small sample size (*N* = 1) precludes it from statistical analyses, however at this point, it differs from *C.rukhadeva* and C.cf.rukhadeva in having 34 PVT versus 26–30; 14 LRT versus 18–20; 172 VSM versus 152–165; 12 FS versus 13–18; 12 PCS versus 13–17 and 1 PCT versus 2 or 3, collectively. From *C.rukhadeva* by having 12 FS versus 16 or 17; and five BB versus three. From *Cyrtodactylusrivularis* sp. nov. it differs in having 14 LRT versus 18–20; 172 VSM versus 160–166; 12 FS versus 14–16; and five BB versus three or four. Discrete differences between *Cyrtodactyluskochangensis* sp. nov. and C.cf.kochangensis sp. nov. and all other species and populations are presented in Tables 4, 5.

##### Natural history.

**Figure 11.**
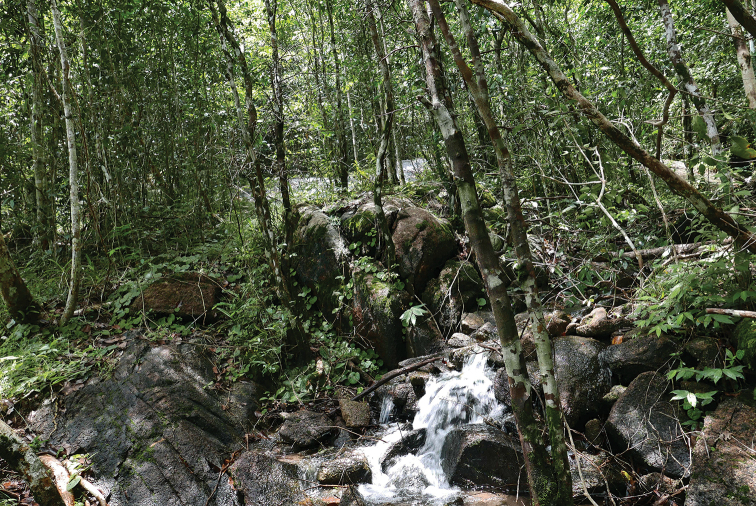
Habitat of *Cyrtodactyluskochangensis* sp. nov. at Thailand, Ranong Province, Mueng Ranong District, Ko Phayam Subdistrict, Ko Chang.

The holotype (ZMKU R 00945) was collected at night (2107 h) among branches of a small tree approximately 100 cm above the ground at 36 m elevation with a temperature of 28.6 °C and relative humidity of 83.9%. The surrounding habitat was dry evergreen forest with a rocky stream nearby (Fig. 11). The new species was found to co-occur with two other species of gekkonid lizards, *Cyrtodactylusoldhami* (Theobald, 1876) and *Gekkotokehos* (Grismer, Wood, Grismer, Quah, Thy, Phimmachak, Sivongxay, Seateun, Stuart, Siler, Mulcahy, Anamza & Brown, 2019).

#### ﻿ Cyrtodactylus uthaiensis sp. nov.

Taxon classificationAnimaliaSquamataGekkonidae

333ED315-A455-5578-9D5E-3A3BC20D8AEB

https://zoobank.org/9422F241-3A46-4FAD-97C4-D6A6AF16B405

**Figure 12.**
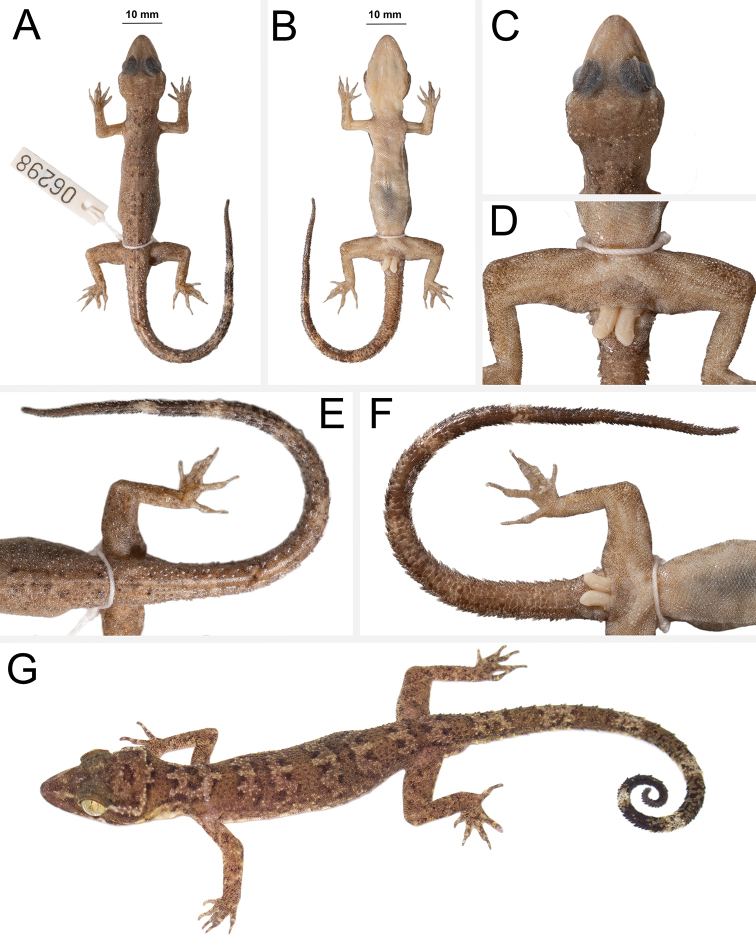
Holotype of *Cyrtodactylusuthaiensis* sp. nov. ZMKU R 00949 (field no. AA 06298) from Thailand, Uthai Thani Province, Lan Sak District, Thung Na Ngam Subdistrict **A** dorsal view **B** ventral view **C** dorsal view of head **D** ventral view of femoral and precloacal regions **E** dorsal view of tail **F** ventral view of tail **G** holotype in life.

Fig. 12 Suggested Common Name: Uthai Thani Bent-toed Gecko

##### Holotype.

Adult male ZMKU R 00949 from Thung Na Ngam Subdistrict, Lan Sak District, Uthai Thani Province, Thailand (15.37649°N, 99.63426°E, 106 m a.s.l.), collected by Attapol Rujirawan, Siriporn Yodthong, Korkhwan Termprayoon, and Natee Ampai on 18 June 2018.

##### Diagnosis.

*Cyrtodactylusuthaiensis* sp. nov. can be separated from all other species of the *brevipalmatus* group by the combination of having 13–15 supralabials, 10–11 infralabials, 33 paravertebral tubercles, 17 rows of longitudinally arranged tubercles, 36 transverse rows of ventrals, 159 longitudinal rows of ventrals, eight expanded subdigital lamellae on the fourth toe, 12 unexpanded subdigital lamellae on the fourth toe, 20 total subdigital lamellae on the fourth toe; seven expanded subdigital lamellae on the fourth finger, 11 unexpanded subdigital lamellae on the fourth finger, 18 total subdigital lamellae on the fourth finger; 16 total enlarged femoral scales, 12 total femoral pores; 14 enlarged pore-bearing precloacals; enlarged femorals and enlarged precloacals not continuous; proximal femorals less than one-half the size of the distal femorals; small tubercles on forelimbs and flanks; large dorsolateral caudal tubercles and wide ventrolateral caudal fringe; ventrolateral caudal fringe composed scales of different size; tail circular in cross-section; slightly enlarged medial subcaudals intermittent, medially furrowed, posteriorly emarginated; maximum SVL 58.1 mm; and six dark transverse body bands (Tables 5, 6).

##### Description of holotype

**(Fig. 12).** Adult male SVL 58.1 mm; head moderate in length (HL/SVL 0.28), width (HW/HL 0.68), depth (HD/HL 0.39), distinct from neck, triangular in dorsal profile; lores concave slightly anteriorly, weakly inflated posteriorly; prefrontal region slightly concave; canthus rostralis rounded; snout elongate (ES/HL 0.40), rounded in dorsal profile; eye large (ED/HL 0.29); ear opening elliptical, small; eye to ear distance greater than diameter of eye; rostral rectangular, dorsally furrowed, bordered posteriorly by large left and right supranasals, bordered laterally by first supralabials; external nares bordered anteriorly by rostral, dorsally by large supranasal, posteriorly by two smaller postnasals, ventrally by first supralabial; 13R/15L rectangular supralabials tapering smoothly to posterior margin of eye; 10R/11L infralabials tapering smoothly to posterior margin of eye; scales of rostrum and lores flat to domed, slightly larger than granular scales on top of head and occiput; scales of occiput intermixed with distinct, small tubercles; superciliaries subrectangular, largest dorsally and anteriorly; mental triangular, bordered laterally by first infralabials and posteriorly by large left and right trapezoidal postmentals contacting medially for approximately 40% of their length posterior to mental; one row of slightly enlarged, elongate sublabials extending posteriorly to fifth(L) and seventh(R) infralabial; gular and throat scales small, granular, grading posteriorly into slightly larger, flatter, smooth, imbricate, pectoral and ventral scales.

**Table 6.** Significant *p*-values from the results of the ANOVA analyses comparing all combinations of OTU pairs of the *Cyrtodactylusbrevipalmatus* group. SVL and TL4U are not listed because no species pairs differed significantly from one another. * = Results based on a Games-Howell *post hoc* test. Character abbreviations are listed in the Materials and methods.

**Morphometric characters**	AG*	HumL*	**ForL**	**FemL**	**TibL**	**HL**	**HW**	HD*	ED*	EE*	**ES**	EN*	**IO**	**EL**	**IN**
*Cyrtodactylusfluvicavus* sp. nov. vs. *C.brevipalmatus*			0.00	0.01		< 0.001	0.001	0.048			< 0.001	0.004		0.002	0.004
*C.interdigitalis* vs. *C.brevipalmatus*			0.00		0.00	0.00					< 0.001				
*C.ngati* vs. *C.brevipalmatus*						0.00						0.003		0.001	< 0.001
*C.ngati*3 vs. *C.brevipalmatus*	0.014			0.03	0.01	< 0.001		0.008		0.038	< 0.001	0.004			0.000
*C.rukhadeva* vs. *C.brevipalmatus*						< 0.001	0.001	0.022			< 0.001	0.021			
*C.* sp.9 vs. *C.brevipalmatus*						0.01	< 0.001				< 0.001	0.03			
*C.interdigitalis* vs. *Cyrtodactylusfluvicavus* sp. nov.					0.01										
*C.ngati* vs. *Cyrtodactylusfluvicavus* sp. nov.	< 0.001	0.002		0.04			< 0.001	0.005			< 0.001			< 0.001	
*C.ngati*3 vs. *Cyrtodactylusfluvicavus* sp. nov.	< 0.001									0.025				0.038	
*C.rukhadeva* vs. *Cyrtodactylusfluvicavus* sp. nov.	0.049		< 0.001	< 0.001										0.004	
*C.* sp.9 vs. *Cyrtodactylusfluvicavus* sp. nov.			0.0					0.007			0.013	0.023		0.007	
*C.ngati* vs. *C.interdigitalis*							0.007		0.031		0.010			< 0.001	0.000
*C.ngati*3 vs. *C.interdigitalis*	0.011														0.003
*C.rukhadeva* vs. *C.interdigitalis*			0.0	0.01	0.00								0.044		
*C.* sp.9 vs. *C.interdigitalis*					0.00		0.007								
*C.ngati*3 vs. *C.ngati*	< 0.001	0.019						0.006			< 0.001			0.001	
*C.rukhadeva* vs. *C.ngati*		0.003					< 0.001	< 0.001	0.046		< 0.001	0.001	< 0.001	< 0.001	0.001
*C.* sp.9 vs. *C.ngati*	< 0.001						< 0.001	0.042			0.007	< 0.001		< 0.001	0.000
*C.rukhadeva* vs. *C.ngati*3	< 0.001	0.021	0.0	0.00						0.007		0.02			0.006
*C.* sp.9 vs. *C.ngati*3	0.001				0.01	0.03	0.003	0.043		0.001	0.019	0.019			0.003
*C.* sp.9 vs. *C.rukhadeva*	0.02	0.004			0.02			0.033							
**Meristic characters**	**SL**	IL*	PVT*	**LRT**	**VS**	**VSM**	**TL4E**	**TL4T**	**FL4E**	FL4U*	FL4T*	**FS**	PCS*	BB*	
*Cyrtodactylusfluvicavus* sp. nov. vs. *C.brevipalmatus*							0.037						< 0.001	0.05	
*C.interdigitalis* vs *C.brevipalmatus*				< 0.001			0.000				0.002		0.044		
*C.ngati* vs *C.brevipalmatus*									< 0.001			0.028	< 0.001		
*C.ngati*3 vs *C.brevipalmatus*				0.017									0.029		
*C.rukhadeva* vs *C.brevipalmatus*				< 0.001									< 0.001		
*C.* sp.9 vs *C.brevipalmatus*				< 0.001	0.003					0.022			< 0.001	0.05	
*C.interdigitalis* vs *Cyrtodactylusfluvicavus* sp. nov.				0.000	0.000	0.021					< 0.001	0.000		< 0.001	
*C.ngati* vs *Cyrtodactylusfluvicavus* sp. nov.	0.037		0.002					< 0.001	< 0.001	0.033		0.000	0.002	< 0.001	
*C.ngati*3 vs *Cyrtodactylusfluvicavus* sp. nov.												0.005			
*C.rukhadeva* vs *Cyrtodactylusfluvicavus* sp. nov.				0.000								0.001		0.001	
*C.* sp.9 vs *Cyrtodactylusfluvicavus* sp. nov.			< 0.001	< 0.001						0.001	0.004	0.020			
*C.ngati* vs *C.interdigitalis*			0.001		0.029			< 0.001	< 0.001	0.029					
*C.ngati*3 vs *C.interdigitalis*					0.029	0.025					0.005				
*C.rukhadeva* vs *C.interdigitalis*		0.004			0.011	0.001	0.044				0.001			0.002	
*C.* sp.9 vs *C.interdigitalis*			0.003		< 0.001	0.005			0.043		0.01			< 0.001	
*C.ngati*3 vs *C.ngati*	0.0267		0.001						< 0.001						
*C.rukhadeva* vs *C.ngati*		< 0.001	< 0.001					0.008	< 0.001	0.008			0.03	< 0.001	
*C.* sp.9 vs *C.ngati*	0.003		0.016	0.011				0.000	< 0.001	< 0.001			< 0.001	< 0.001	
*C.rukhadeva* vs *C.ngati*3															
*C.* sp.9 vs *C.ngati*3			0.001	0.042						< 0.001	0.001				
*C.* sp.9 vs *C.rukhadeva*	0.029		< 0.001		0.002									0.001	

Body relatively short (AG/SVL 0.46) with well-defined ventrolateral folds; dorsal scales small, granular, interspersed with larger, conical, semi-regularly arranged, weakly keeled tubercles; tubercles extend from occipital region onto base of tail and slightly beyond as paravertebral rows; smaller tubercles extend anteriorly onto nape and occiput, diminishing in size anteriorly; approximately 17 longitudinal rows of tubercles at midbody; approximately 33 paravertebral tubercles; small tubercles on flanks; 36 longitudinal rows of flat, imbricate, ventral scales much larger than dorsal scales; 159 transverse rows of ventral scales; 16 total large femoral scales; 12 total femoral pores; 14 enlarged pore-bearing precloacals; no deep precloacal groove or depression; and two rows of post-precloacal scales on midline.

Forelimbs moderate in stature, relatively short (ForL/SVL 0.14); granular scales of forearm slightly larger than those on body, interspersed with large tubercles; palmar scales rounded, slightly raised; digits well-developed, relatively short, inflected at basal interphalangeal joints; digits narrower distal to inflections; subdigital lamellae wide, transversely expanded proximal to joint inflections, narrower transverse lamellae distal to joint inflections; claws well-developed, claw base sheathed by a dorsal and ventral scale; 7R/7L expanded and 11R/11L unexpanded lamellae beneath the fourth finger; hind limbs larger and thicker than forelimbs, moderate in length (TibL/SVL 0.14), covered dorsally by granular scales interspersed with moderately sized, conical tubercles dorsally and posteriorly and anteriorly by flat, slightly larger, subimbricate scales; ventral scales of thigh flat, subimbricate, larger than dorsals; subtibial scales flat, imbricate; one row of 6R/6L of enlarged pore-bearing femoral scales not continuous with enlarged pore bearing precloacal scales, terminating distally at knee; 8R/8L enlarged femoral scales; proximal femoral scales smaller than distal femorals, the former forming an abrupt union with much smaller, rounded, ventral scales of posteroventral margin of thigh; plantar scales flat; digits relatively long, well-developed, inflected at basal interphalangeal joints; 8R/(broken)L wide, transversely expanded subdigital lamellae on fourth toe proximal to joint inflection that extend onto sole, 12R/(broken)L unexpanded lamellae beneath first toe; and claws well-developed, sheathed by a dorsal and ventral scale at base.

Tail original, long 76.7 mm (TL/SVL 1.32), 4.1 mm in width at base, tapering to a point; sub-circular or nearly round in cross-section; dorsal scales flat, square bearing tubercles forming paravertebral rows and large tubercles forming a dorsolateral longitudinal row; slightly larger, posteriorly directed, semi-spinose tubercles forming small but distinct ventrolateral caudal fringe; larger scales of ventrolateral fringe occur at regular intervals; slightly enlarged medial subcaudals intermittent, medially furrowed, posteriorly; single enlarged medial subcaudals absent; subcaudal scales, larger than dorsal caudal scales; base of tail bearing hemipenial swellings; 3R/3L conical postcloacal tubercles at base of hemipenial swellings; and postcloacal scales flat, imbricate.

##### Coloration in life

**(Fig. 12).** Ground color of the head, body, limbs, and tail pale-brown; dark, diffuse mottling on interorbital region and snout; dark blotch on top of head; wide, pale-colored postorbital stripe irregularly edged in dark brown extends from posterior margin of one eye across nape to posterior margin of other eye; ventral portion of lores, suborbital region, and supralabials darkly mottled; wide, dark brown nuchal band, bearing two posterior projections; six irregularly shaped darkly edged body bands extending between forelimb and hind limb insertions followed by one dark sacral band; paired dark brown paravertebral blotches on nape; band interspaces bearing irregularly shaped, dark-colored markings; dark-colored speckling on limbs and digits; digits bearing pale-colored bands; seven wide dark-colored caudal bands separated by six pale-colored bands; caudal bands encircle tail resulting in heavily mottled subcaudal region; all other ventral surfaces beige, generally immaculate; and iris gold in color.

##### Distribution.

*Cyrtodactylusuthaiensis* sp. nov. is currently known from the type locality at Thung Na Ngam Subdistrict, Lan Sak District, Uthai Thani Province, Thailand.

##### Etymology.

The specific epithet *uthaiensis* refers to the type locality, Uthai Thani Province, Thailand.

##### Comparisons.

*Cyrtodactylusuthaiensis* sp. nov. is the sister species to a clade comprised the sister species *C.interdigitalis* and *C.* sp.11 (Fig. 2). Together, these taxa form the sister lineage to C.cf.ngati1, C.cf.ngati2, and all other *C.ngati*. *Cyrtodactylusuthaiensis* sp. nov. differs from those lineages by an uncorrected pairwise sequence divergence of 5.81–8.13% (Table 2). We are aware that any comparison based on morphometric and meristic characters are preliminary being that there is only one sample of *Cyrtodactylusuthaiensis* sp. nov. and that additional sampling may preclude some characters and being diagnostic just as it may reveal that other characters are diagnostic (see Table 5). Therefore, at this point we rely on the invariable categorical characters to separate *Cyrtodactylusuthaiensis* sp. nov. from other species in the *brevipalmatus* group. *Cyrtodactylusuthaiensis* sp. nov. differs from *C.brevipalmatus*, *Cyrtodactylusfluvicavus* sp. nov., *C.interdigitalis*, *C.ngati*, *C.ngati*3, C.cf.ngati1, C.cf.ngati2, *C.rukhadeva*, C.cf.rukhadeva and *C.* sp.13 by having large dorsolateral caudal tubercles (DCT) forming a wide ventrolateral caudal fringe (VLF1). *Cyrtodactylusuthaiensis* sp. nov. is further differentiated from *C.ngati*3, C.cf.ngati1, C.cf.ngati2, *C.interdigitalis*, *C.rukhadeva*, C.cf.rukhadeva, and sp.13 by having a ventrolateral fringe not homogenous (VLF2). It differs from *Cyrtodactyluskochangensis* sp. nov., *Cyrtodactylusrivularis* sp. nov., *C.rukhadeva*, C.cf.rukhadeva, and *C.* sp.11 by having tail that is more circular in cross-section than square (TLcross). From *Cyrtodactylusrivularis* sp. nov., *C.rukhadeva* and C.cf.rukhadeva, it differs by having enlarged, unmodified, medial subcaudal scales (SC1). From *Cyrtodactylusrivularis* sp. nov., *C.rukhadeva* and C.cf.rukhadeva, it differs by lacking single, enlarged, medial subcaudal scales (SC2). From all species in the *brevipalmatus* group except *C.interdigitalis* it differs by having posteriorly emarginated, medial subcaudals bearing a median furrow (SC3).

##### Natural history.

**Figure 13.**
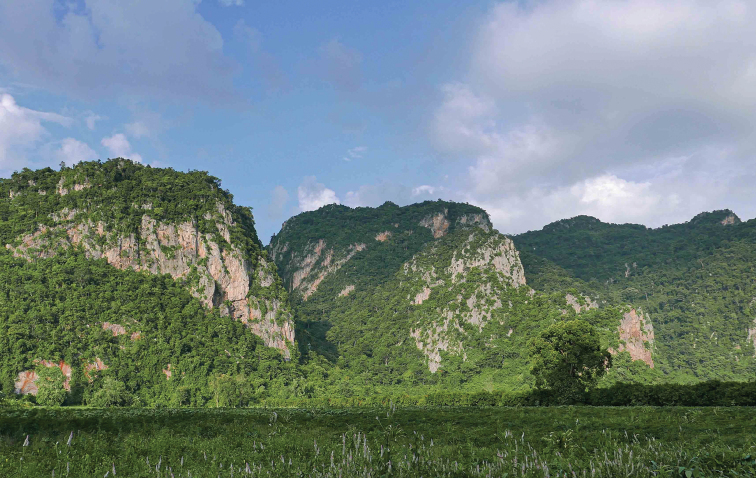
Habitat of *Cyrtodactylusuthaiensis* sp. nov. at Thailand, Uthai Thani Province, Lan Sak District, Thung Na Ngam Subdistrict.

*Cyrtodactylusuthaiensis* sp. nov. is the only species of the *brevipalmatus* group that occurs in an isolated hilly area within the Chao Phraya River Basin (Fig. 1). The holotype (ZMKU R 00949) was collected at night (2055 h) on a bamboo twig approximately 170 cm above ground level at 106 m elevation. The habitat was isolated karst formations within a mixed deciduous forest. This area was surrounded by agricultural fields (plantations and rice fields) and human residential areas (Fig. 13). The new species was found to co-occur with a gekkonid lizard, *Dixoniussiamensis* (Boulenger, 1899).

**Table 7.** Summary statistics from the PERMANOVA analysis of the OTUs and proposed morphogroups of the *Cyrtodactylusbrevipalmatus* group.

OTU pairs	F model	R^2^	*p*-value	*p*-adjusted
*C.rukhadeva* vs. C.cf.ngati2	6.8474	0.4064	0.015	0.544
*C.rukhadeva* vs. *C.ngati*3	8.8824	0.4467	0.003	0.122
*C.rukhadeva* vs. *C.interdigitalis*	3.3630	0.2189	0.006	0.201
*C.rukhadeva* vs. *C.ngati*	10.4580	0.4874	0.003	0.114
*C.rukhadeva* vs. *C.brevipalmatus*	6.5983	0.3367	0.000	0.012
*C.rukhadeva* vs. *Cyrtodactylusfluvicavus* sp. nov.	6.6357	0.3067	0.000	0.004
*C.rukhadeva* vs. *C.* sp.9	3.8646	0.1945	0.001	0.033
C.cf.ngati2 vs. *C.brevipalmatus*	15.4818	0.7559	0.048	1.000
C.cf.ngati2 vs. *Cyrtodactylusfluvicavus* sp. nov.	15.9186	0.6946	0.027	0.967
C.cf.ngati2 vs. *C.* sp.9	19.0130	0.7038	0.022	0.804
*C.ngati*3 vs. *C.interdigitalis*	4.4753	0.4723	0.029	1.000
*C.ngati*3 vs. *C.brevipalmatus*	14.9425	0.7135	0.018	0.643
*C.ngati*3 vs. *Cyrtodactylusfluvicavus* sp. nov.	8.7953	0.5237	0.009	0.317
*C.ngati*3 vs. *C.* sp.9	14.7978	0.6218	0.006	0.226
*C.interdigitalis* vs. *C.ngati*	9.8976	0.6644	0.029	1.000
*C.interdigitalis* vs. *C.brevipalmatus*	4.5646	0.3947	0.008	0.278
*C.interdigitalis* vs. *Cyrtodactylusfluvicavus* sp. nov.	6.7120	0.4272	0.003	0.124
*C.interdigitalis* vs. *C.* sp.9	5.6585	0.3614	0.002	0.067
*C.ngati* vs. *C.brevipalmatus*	7.4818	0.5550	0.018	0.643
*C.ngati* vs. *Cyrtodactylusfluvicavus* sp. nov.	22.8234	0.7405	0.008	0.283
*C.ngati* vs. *C.* sp.9	17.0146	0.6540	0.006	0.227
*C.brevipalmatus* vs. *Cyrtodactylusfluvicavus* sp. nov.	17.8585	0.6410	0.001	0.048
*C.brevipalmatus* vs. *C.* sp.9	9.3960	0.4607	0.001	0.025
*Cyrtodactylusfluvicavus* sp. nov. vs. *C.* sp.9	8.2047	0.3869	0.000	0.005

## ﻿Discussion

**Figure 14.**
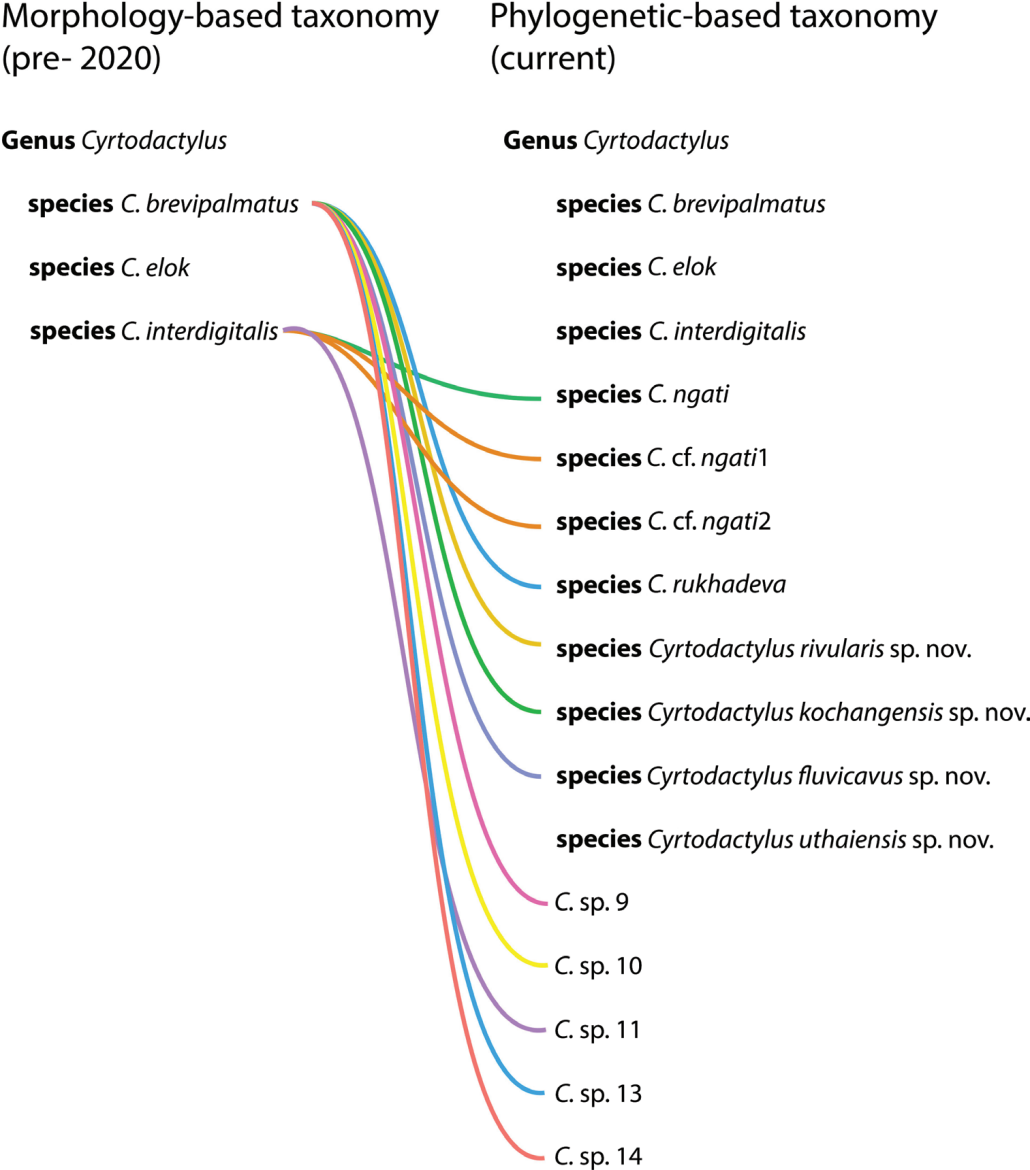
Comparison of a morphology-based taxonomy pre-2020 and the current phylogenetic-based taxonomy illustrating the misrepresentation of the diversity of the *Cyrtodactylusbrevipalmatus* group by the former. Colored lines connecting species on the left to species on the right indicate under which name the former were masquerading as the latter.

A taxonomy consistent with evolutionary history is paramount to any downstream comparative analyses employed to address the evolution of other features of the group be they ecology, behavior, or habitat preference. By delimiting new and existing species using a phylogenetic analysis and then diagnosing those species using univariate and multivariate statistical analyses of their morphological data, phylogenetic history will not conflated with convergent evolution (Fig. 14). This is extremely important for conservation and natural resource management programs so that efforts can be vectored towards all recognized species without missing the species that were previously masquerading among the synonymies of an erroneous taxonomy. This is especially true for range-restricted, highly specialized species, whose general morphological similarity often does not align with their phylogenetic history as exemplified in the *brevipalmatus* group (Fig. 3). Given that upland tropical regions are currently some of the most vulnerable to climate change (Gehrig-Fasel et al. 2007; Myers-Smith et al. 2011; Scridel et al. 2018), this argument becomes especially germane for the entire *brevipalmatus* group— the majority of whose species are upland populations and potentially range-restricted.

The description of the four new species, *Cyrtodactylusfluvicavus* sp. nov., *Cyrtodactyluskochangensis* sp. nov., *Cyrtodactylusrivularis* sp. nov., and *Cyrtodactylusuthaiensis* sp. nov. adds to the rapidly growing list of new species of *Cyrtodactylus* from Thailand. Many of the poorly sampled populations reported here such as *C.* sp.9 from Thong Pha Phum National Park, Kanchanaburi Province; *C.* sp.10 from Chao Doi Waterfall, Tak Province; *C.* sp.11 from Phu Hin Rong Kla National Park, Phitsanulok Province; *C.* sp.13 from Ban Saphan Lao, Kanchanaburi Province and Thung Yai Naresuan Wildlife Sanctuary, Tak Provinces; *C.* sp.14 from Langkawi Island, Peninsular Malaysia; C.cf.ngati1 and C.cf.ngati2 from Xaignabouli and Vientiane Provinces, respectively, Laos (Figs 1, 2), will likely be described as new species when new material becomes available. Additionally, many populations listed in various non-technical websites as *C.interdigitalis* or *C.brevipalmatus* based on overall similarity or general distribution, may also be recovered as new species or delegated to some of the more recently described species. This research continues to highlight the unrealized biodiversity in climatically imperiled upland ecosystems throughout Southeast Asia.

## Supplementary Material

XML Treatment for Cyrtodactylus fluvicavus

XML Treatment for Cyrtodactylus rivularis

XML Treatment for Cyrtodactylus kochangensis

XML Treatment for Cyrtodactylus uthaiensis

## Supplementary materials

Supplementary material 1 Data frame for the multiple factor analysis of the putative species of the *Cyrtodactylusbrevipalmatus* group Data type morphological data Explanation note Data frame for the multiple factor analysis of the putative species of the *Cyrtodactylusbrevipalmatus* group. https://binary.pensoft.net/file/769836This dataset is made available under the Open Database License (http://opendatacommons.org/licenses/odbl/1.0/). The Open Database License (ODbL) is a license agreement intended to allow users to freely share, modify, and use this Dataset while maintaining this same freedom for others, provided that the original source and author(s) are credited.L. Lee Grismer, Anchalee Aowphol, Siriporn Yodthong, Natee Ampai, Korkhwan Termprayoon, Akrachai Aksornneam, Attapol Rujirawan
